# Investigation of Electronic Structures of Triplet States Using Step-Scan Time-Resolved Fourier-Transform Near-Infrared Spectroscopy

**DOI:** 10.1021/acs.jpclett.3c03521

**Published:** 2024-01-19

**Authors:** Chia Chun Wu, Yu-Xiang Tsai, Li-Kang Chu, I-Chia Chen

**Affiliations:** Department of Chemistry, National Tsing Hua University, Hsinchu, Taiwan 300044, Republic of China

## Abstract

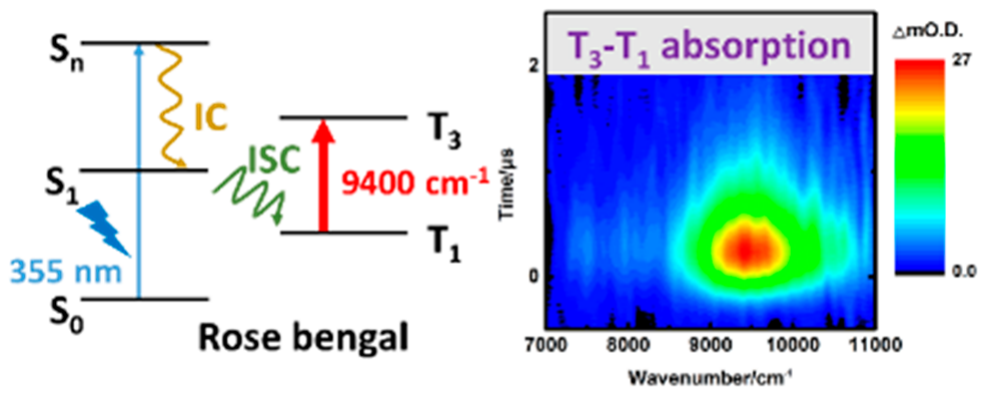

Triplet transitions of light-emitting materials, including rose bengal, tris(2-phenylpyridine)iridium(III) [Ir(ppy)_3_], tris(1-phenylisoquinoline)iridium(III) [Ir(piq)_3_], and bis[2-(4,6-difluorophenyl)pyridinato-C2,N](picolinato)iridium(III) (FIrpic), were studied using step-scan time-resolved Fourier-transform near-infrared spectroscopy. The samples were excited to their singlet excited states by a 355 nm laser and then underwent efficient conversions/crossings to their triplet manifolds. For rose bengal, a transient absorption band appeared at 9400 cm^–1^, attributed to the T_3_ ← T_1_ transition based on the corresponding time evolution and the theoretical calculations. For Ir(ppy)_3_, Ir(piq)_3_, and FIrpic, the most intense bands were observed at 7700, 7500, and 7500 cm^–1^ and assigned to T_7_ ← T_1_, T_6_ ← T_1_, and T_6_ ← T_1_ transitions, respectively. For Ir(ppy)_3_, the most intense band involved transitions between different triplet metal-to-ligand charge transfer (^3^MLCT) states, while for Ir(piq)_3_ and FIrpic, they involved a metal center to ^3^MLCT transition. These T_1_ states were assigned to ^3^MLCT.

Applications of triplet excited states in photocatalysis, photovoltaics, biological imaging, light-sensitive materials for detecting singlet oxygen, and thermally activated delayed fluorescence (TADF) have attracted a great deal of attention.^1−11^ Miscellaneous experimental methods, including pump–probe femtosecond transient absorption,^12−16^ nanosecond transient absorption,^17−21^ time-resolved fluorescence up-conversion,^20^ time-correlated single-photon counting (TCSPC),^13,15,17,20,22,23^ time-resolved photoluminescence,^14−19,21^ and theoretical methods, such as time-dependent density functional theory (TD-DFT),^12−18,20,22,24^ have been extensively employed to study the radiative/nonradiative dynamics and kinetics of the triplet excited states of the aforementioned materials. Understanding the energy flows and energetics is essential for the utilization of excitonic energy in radiative processes. However, time-resolved luminescence spectroscopic methods cannot provide direct evidence of the dark states or the interconversion of these states.^19^ Moreover, understanding the high-lying triplet excited states might facilitate the energy utilization of the ladder-like energy-relaying exciplex in TADF materials.^16^ Unfortunately, the energetics and dynamics of the high-lying triplet excited states have remained less studied because their energy gaps lie mostly in the infrared and near-infrared regions, which are not easily accessed by means of the conventional time-resolved techniques.

In this work, a step-scan time-resolved Fourier-transform near-infrared (FTNIR) spectrometer, which can be operated in absorption and emission modes to detect transient species on the nanosecond to microsecond time scale,^25^ was employed to detect the long-lived triplet intermediates. We report the spectroscopic and kinetic results on the triplet states of the following molecular systems: 4,5,6,7-tetrachloro-2′,4′,5′,7′-tetraiodofluorescein [rose bengal (RB)] and three iridium complexes, tris(2-phenylpyridine)iridium(III) [Ir(ppy)_3_], tris(1-phenylisoquinoline)iridium(III) [Ir(piq)_3_], and bis[2-(4,6-difluorophenyl)pyridinato-C2,N](picolinato)iridium(III) (FIrpic). Their molecular structures are shown in Figures 1a–4a. These iridium complexes are organic light-emitting diode (OLED) materials covering a wide range of emission wavelengths, and each has a nearly 100% intersystem crossing (ISC) efficiency.^26−28^ They exhibited the triplet states of possibly different characters: triplet metal-to-ligand charge transfer (^3^MLCT) and triplet inter- or intraligand-centered (^3^LL′C or ^3^LC) states. The goal of this work was to identify these triplet states and to gain insights into their electronic properties using the time-resolved FTNIR technique, which has the advantages of a wide energy range of detection windows and temporal resolution.

**Figure 1:**
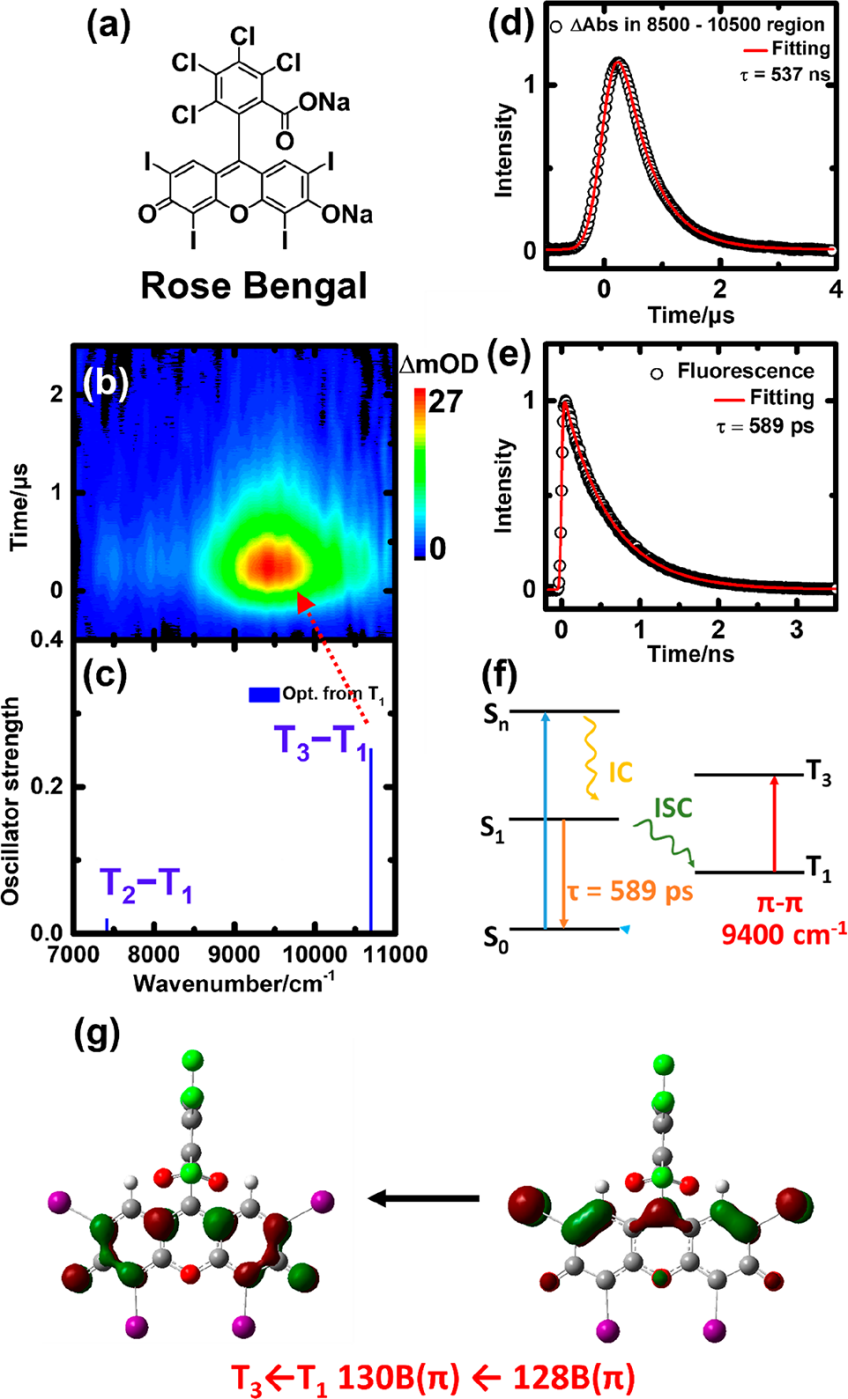
(a) Molecular structure of RB. (b) Transient absorption of 0.1 mM RB in methanol excited at 355 nm. (c) Transitions of TD-DFT calculations using optimized geometries for the T_1_ (blue line) state (B3LYP/6-311+G*/LanL2DZ). (d) Integrated transient absorption at 8500–10500 cm^–1^ region plotted vs time. The fitted curve is the convoluted single-exponential decay function with a Gaussian instrument response fwhm of 400 ns. The best-fit decay time constant (τ) is 537 ns. (e) Fluorescence decay of the RB S_1_ state measured by time-correlated single-photon counting and a fitted decay time constant of 589 ps. (f) Schematic diagram of the formation and transition of triplet RB. (g) MOs of the T_3_–T_1_ transition.

The time-resolved NIR absorption spectra of 0.1 mM RB in methanol upon 355 nm light excitation are shown in Figure 1b. An intense absorption band emerged at ∼9400 cm^–1^, with a full width at half-maximum (fwhm) of ∼1000 cm^–1^. After integration of the absorption difference in the 8500–10500 cm^–1^ region versus time, the decay curve, shown in Figure 1d, was deconvoluted with an instrument response function (fwhm of 400 ns, limited by the preamplifier) to yield a decay time constant of 537 ns. This band position agrees with that observed by Larkin et al.,^29^ and the decay mainly results from oxygen quenching; the decay time constant is close to the decay of the triplet RB reported by Tsimvrakidis et al.^30^ This band is blue-shifted to 9660 and 9850 cm^–1^ in the less polar solvents acetonitrile (ACN) and THF, respectively, with decay time constants of 700 and 1190 ns, respectively. The experimental data are listed in Figure S3. This implies that the upper electronic state is more polar to improve the solvation stability to display a red-shifted transient band in more polar solvents. The lifetimes of the triplet states mainly resulted from oxygen quenching in solution. Singlet oxygen formed from the reaction of triplet RB reacted with THF to produce tetrahydrofuran-2(3*H*)-one and the byproduct 2-hydroperoxyl-THF.^31^ Therefore, the oxygen was consumed in a sealed sample cell after some time; a relatively longer lifetime of triplet RB was observed in THF. We also measured the decay of the transient under the degassed condition, and the lifetime was measured to be 13 and 26 μs in 1 and 0.1 mM methanol solutions, respectively. In nitrogen-saturated ACN solution, the lifetime was reported to be 140 μs versus 100 μs in aqueous solution.^32^

From the DFT calculations, each vertical T_*n*_–S_0_ transition energy was obtained and the energy differences between T_*n*_ and T_1_ were estimated by subtracting the transition energy of the T_1_–S_0_ transition from that of the T_*n*_–S_0_ transition. Those values are listed in Table 1. Only the energy differences in the T_2_–T_1_ and T_3_–T_1_ transitions at 7289 and 9596 cm^–1^, respectively, lie in our detection window. The transition from T_1_ to T_2_ corresponds to an electron moving from the molecular orbital (MO) 129(π_2_) to 130(π_1_), where 130(π_1_) is the highest occupied MO (HOMO) and 129(π_2_) is the second highest occupied MO (SOMO). The T_3_–T_1_ transition corresponds to the 130(π_1_) ← 127(n+π_4_) transition. The calculated electron configurations of these states are shown in Figure S4.

**Table 1:** Energies of the Vertical Transition from the Optimized S_0_ and T_1_ Structures of RB to the T_*n*_ States and Energy Differences between T_*n*_ and T_1_, Calculated Using B3LYP/6-311+G*/LanL2DZ

upper state	T_*n*_–S_0_ (eV)^a^	T_*n*_–T_1_ (cm^–1^)^a^	T_*n*_–T_1_ (eV)^b^^,^^c^	T_*n*_–T_1_ (cm^–1^)^b^
T_1_	1.6253	–	–	–
T_2_	2.5290	7289	0.919 (0.0202)	7412
T_3_	2.8150	9596	1.326 (0.2522)	10695
T_4_	3.0982	11880	1.412 (0.0009)	11388
T_5_	3.1827	12561	1.4543 (0.0005)	11730
T_6_	3.1994	12696	1.5643 (0.0001)	12617
T_7_	3.2036	12730	1.5831 (0.019)	12769

a From the optimized S_0_ structure.

b From the optimized T_1_ structure.

c Oscillator strength in parentheses.

On the basis of the optimized T_1_ geometry, the vertical transition energies and oscillator strengths (*f*) of the T_*n*_–T_1_ transition are listed in Table 1. Two bands at 7412 cm^–1^ (T_2_–T_1_; *f* = 0.0202) and 10695 cm^–1^ (T_3_–T_1_; *f* = 0.2522) are within the detection window, as shown in the stick plot in Figure 1c. The oscillator strength of the T_3_–T_1_ transition is 10 times larger than that of the other; hence, we assigned the observed 9000–10000 cm^–1^ band to this transition. The calculated band position deviates by ∼14% from the experimental value (in a methanol solvent), and the involved MOs are 130B(π)–128B(π), as shown in Figure 1g. In addition, the T_2_–T_1_ transition either is too weak to be observed or lies below the detection window. Hence, only one electronic band was observed experimentally. In addition, the calculated dipole of T_3_ was 11.7 D versus 10.7 D in T_1_, in agreement with the experimental findings that the upper state is more polar.

Upon excitation with 355 nm light, RB was excited from a nonbonding orbital to a π* orbital. It subsequently underwent internal conversion (IC) to the S_1_ state, followed by efficient ISC to T_1_. The fluorescence emission was detected by TCSPC (experimental details in the Supporting Information) and has a decay lifetime of 589 ps, as shown in Figure 1e; a rapid ISC rate was observed. Accordingly, the transient T_1_ state detected by the FTNIR spectrometer had an IRF-limited rise before returning to the ground state mainly by oxygen quenching with a lifetime of 537 ns (in a methanol solvent). The kinetic model for this energy relaxation process is displayed in Figure 1f.

The time-resolved NIR transient absorption spectra of 1 mM Ir(ppy)_3_ in THF are shown in Figure 2b. An intense band appears at 7700 cm^–1^, with a fwhm of ∼1200 cm^–1^, and a weak band is sporadically visible in the higher-wavenumber range of 8500–10000 cm^–1^. The absorption differences within the wavenumber ranges of 7200–8400 and 8500–10000 cm^–1^ were integrated and plotted versus time and featured a similar decay lifetime of ∼1.6 μs; one of these curves is shown in Figure 2d. This implies that both bands are transitions from a common lower energy level. The green phosphorescence of Ir(ppy)_3_ was measured to yield a lifetime of ∼1.5 μs, as shown in Figure 2e. Iridium complexes undergo very efficient ISC to the triplet manifold and then radiatively relax from lowest triplet state T_1_ to S_0_.^26−28^ Singlet oxygen formed from the reaction of triplet Ir(ppy)_3_ and reacted with THF to create a nearly oxygen-free environment such that the decay of triplet Ir(ppy)_3_ occurred mainly via a radiative process. Because of the agreement between the lifetime of the transient species and the phosphorescence decay, we assigned the transient intermediate to the T_1_ state.

**Figure 2:**
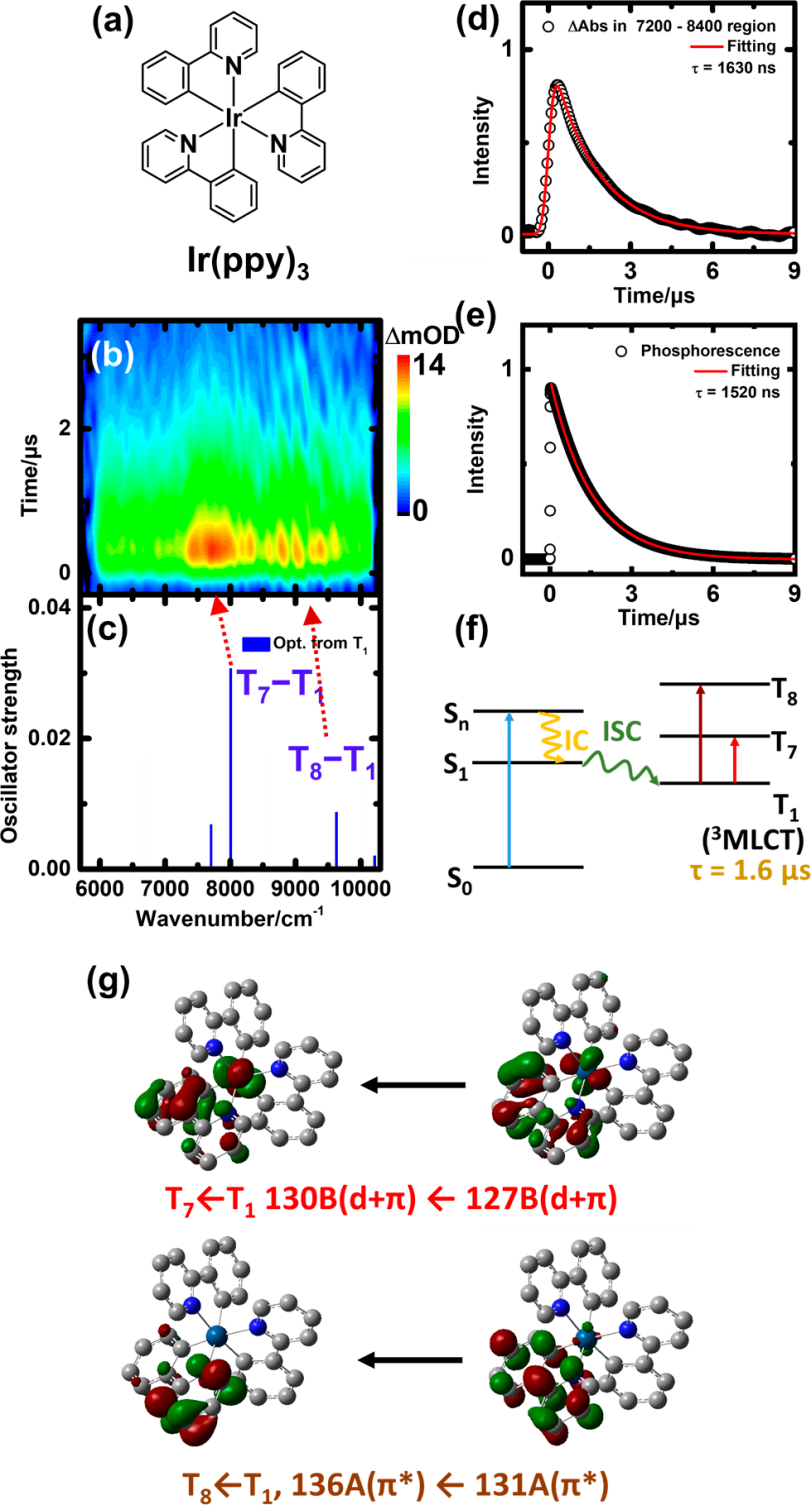
(a) Molecular structure of Ir(ppy)_3_. (b) Transient absorption of 1 mM Ir(ppy)_3_ in THF excited at 355 nm. (c) Transitions of TD-DFT calculations using optimized geometries for the T_1_ (blue line) state (B3LYP/6-311+G*/LanL2TZf). (d) Integrated transient absorption in the range of 7200–8400 cm^–1^ plotted vs time. The fitted curve is convoluted via the single-exponential decay function with a Gaussian instrument response fwhm of 400 ns. The best-fit decay time constant (τ) is 1.6 μs. (e) Phosphorescence decay and a fitted decay time constant of 1.52 μs. (f) Schematic diagram of the formation, decay, and transitions of triplet Ir(ppy)_3_. (g) MOs of the T_7_–T_1_ and T_8_–T_1_ transitions.

In the detection window, TD-DFT calculations yielded six T_*n*_–T_1_ transitions from the optimized S_0_ geometry. Their energies for T_11_–T_16_ relative to T_1_ are 6694, 6775, 8100, 8550, 10082, and 10163 cm^–1^, respectively. Comparison of the calculated energies with the experimental data is insufficient to assign those bands. The T_1_-optimized geometry was then calculated and used to estimate the oscillator strengths of the T_*n*_ ← T_1_ transitions. The optimized T_1_ geometry loses its *C*3 symmetry; the Ir–N and Ir–C bond lengths differed in three ppy ligands, resulting in the splitting of the original degenerate levels. The calculated transition energies are plotted in Figure 2c. The transitions at 7704, 8005, 9623, and 10215 cm^–1^ corresponded to T_6_ ← T_1_ (134A ← 131A; *f* = 0.0068), T_7_ ← T_1_ (130B ← 127B; *f* = 0.0307), T_8_ ← T_1_ (136A ← 131A; *f* = 0.0087), and T_9_ ← T_1_ (135A ← 131A; *f* = 0.0020), respectively; A and B refer to the α and β spin states, respectively. Accordingly, we assigned the intense experimental band centered at 7700 cm^–1^ to the T_7_ ← T_1_ transition and the weak absorption band at 8500–9600 cm^–1^ to the T_8_ ← T_1_ transition. From the calculations, T_6_, T_8_, and the T_9_ ← T_1_ transition primarily involve ppy π ← π transitions and are denoted as ^3^LC transitions. The intense vertical T_7_ ← T_1_ transition is the ^3^(MLCT)_*n*_ ← ^3^(MLCT)_1_ transition, involving electronic transitions between different iridium d orbitals mixed with ppy π orbitals. The MOs involved in the transitions are listed in Figure 2g. Because the calculated vertical transitions using the optimized T_1_ state yielded more accurate band positions and also reliable oscillator strengths, for the next two iridium complexes we report only the calculated vertical transitions based on the optimized T_1_ geometries.

The kinetic model for the relaxation process is briefly summed in Figure 2f. Ir(ppy)_3_ was excited from a nonbonding Ir d orbital to a ppy π* orbital and subsequently underwent internal conversion/ISC to the lowest triplet state. Only one triplet lower state is identified, and it is assigned to the T_1_ (^3^MLCT)_1_ state. This triplet state has a lifetime of 1.5 μs and primarily decays via a radiative process. Two triplet upper states, T_7_ and T_8_, are identified.

The transient difference absorption spectra of 570 μM Ir(piq)_3_ in THF upon 355 nm excitation are shown in Figure 3b. The energy region above 8200 cm^–1^ experienced interference by phosphorescence emission; hence, the signal in the high-wavenumber region was blocked by optical filters. An intense absorption band was observed at ∼7500 cm^–1^. Scattered absorption bands were also observed at 6000–7100 cm^–1^. The decays of absorption at 7100–8100 and 6000–7100 cm^–1^ display similar decay constants of ∼1.1 μs. One of the curves is shown in Figure 3d. The phosphorescence of Ir(piq)_3_ from the radiative relaxation from T_1_ to S_0_ has been reported previously.^27^ The observed phosphorescence lifetime of 1.14 μs closely matches the lifetimes of the absorption bands; hence, they originated from the same state, T_1_.

**Figure 3:**
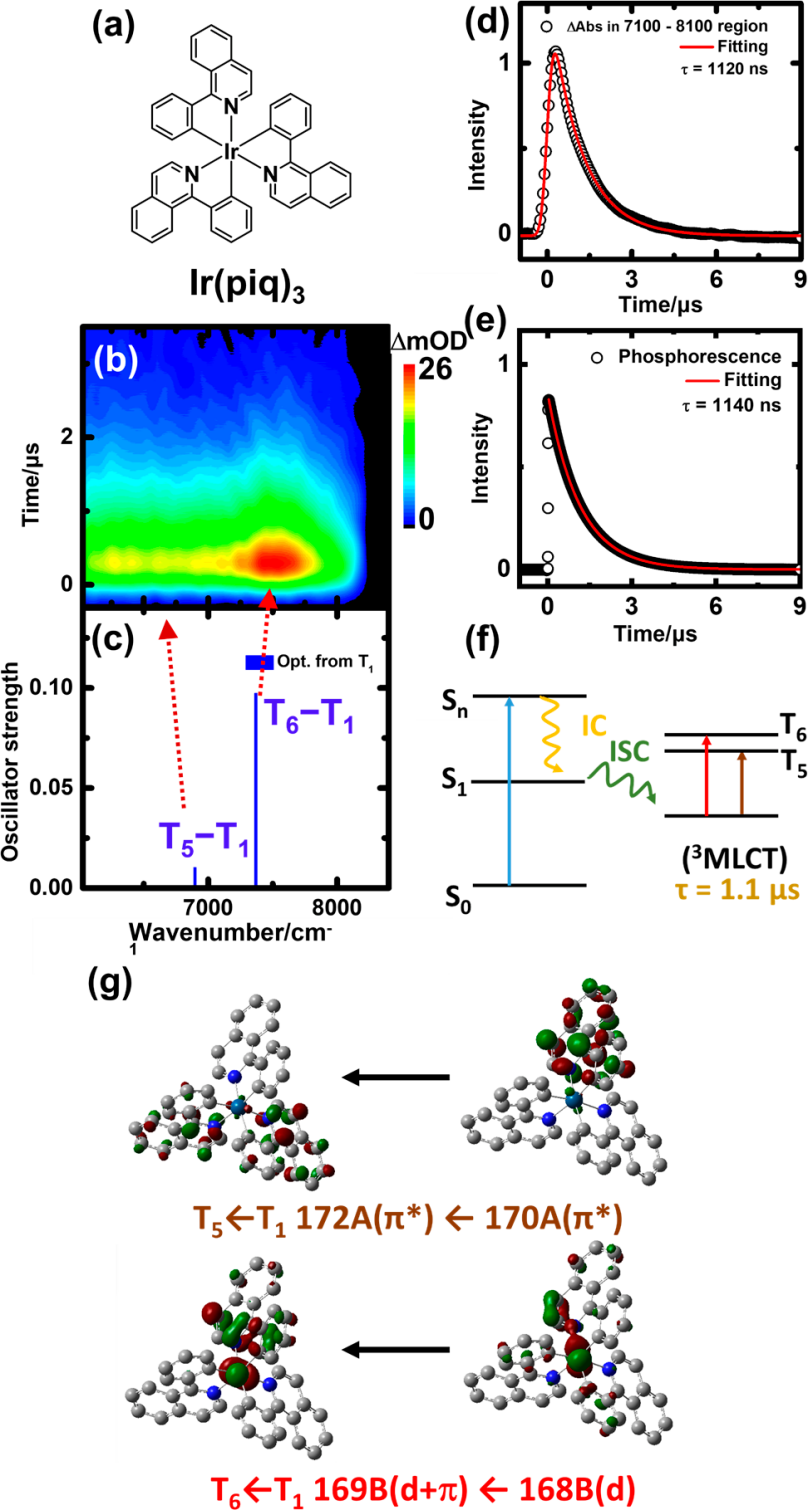
(a) Molecular structure of Ir(piq)_3_. (b) Transient absorption of 570 μM Ir(piq)_3_ in THF excited at 355 nm. (c) Transitions of TD-DFT calculations using optimized geometries for the T_1_ (blue line) state (B3LYP/6-311+G*/LanL2TZf). (d) Integrated transient absorption in the range of 7100–8100 cm^–1^ plotted vs time. The fitted curve is the convoluted single-exponential decay function with a Gaussian instrument response fwhm of 400 ns. The best-fit decay time constant (τ) is 1.1 μs. (e) Phosphorescence decay and a fitted decay time constant of 1.1 μs. (f) Schematic diagram of formation, decay, and transitions of triplet Ir(piq)_3_. (g) MOs of T_5_–T_1_ and T_6_–T_1_ transitions.

From the calculated T_1_ geometry, two transitions at 6896 and 7370 cm^–1^, corresponding to the T_5_ ← T_1_ (172A ← 170A; *f* = 0.0102) and T_6_ ← T_1_ transitions (169B ← 168B; *f* = 0.0973), respectively, are obtained, and their positions are plotted in Figure 3c. Their involved MOs are shown in Figure 3g. From their wavenumber positions and oscillator strengths, we assigned the observed band at 7500 cm^–1^ to the T_6_ ← T_1_ transition and the weak band at 6000–7100 cm^–1^ to the T_5_ ← T_1_ transition. T_5_ ← T_1_ and T_6_ ← T_1_ transitions involved the interligand π* ← π* and d+π ← d transitions, respectively. Therefore, they are denoted as an interligand-centered triplet to triplet transition (^3^LL′C) and a metal-centered to MLCT transition (^3^MLCT ← ^3^MC), respectively. Concomitantly, the kinetic model for the relaxation process of Ir(piq)_3_ is shown in Figure 3f. The relaxation of the excited state is similar to that of Ir(ppy)_3_; the short-lived lowest triplet state is a ^3^MLCT state and primarily undergoes a radiative process to relax its energy.

The time-resolved absorption spectra of 1 mM FIrpic in THF are shown in Figure 4b. An intense absorption band is observed at 6200–8200 cm^–1^, with a weak band appearing at 8600–10000 cm^–1^. Both decays of absorption in these two regions are ∼1.5 μs; one of the decay curves is plotted in Figure 4d. The emission of FIrpic in the visible light range was measured and fitted with a single-exponential decay lifetime of ∼1.5 μs, as shown in Figure 4e. This emission was assigned to phosphorescence of T_1_.^28^ From the same temporal behavior, all of the phosphorescence and transient absorption bands originated from the T_1_ state.

**Figure 4:**
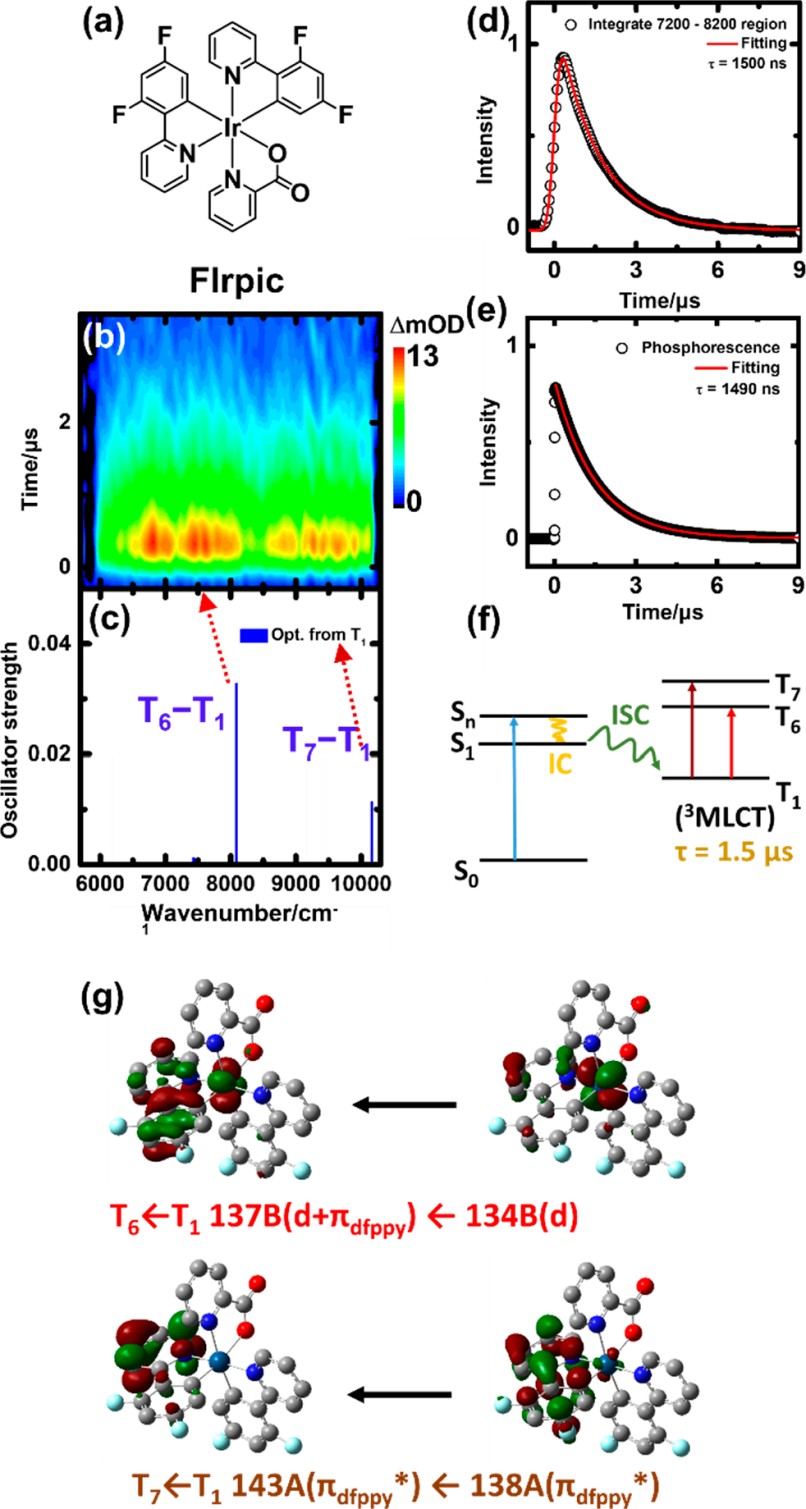
(a) Molecular structure of FIrpic. (b) Transient absorption of 1 mM FIrpic in THF excited at a wavelength of 355 nm. (c) Transitions of TD-DFT calculations using optimized geometries for the T_1_ (blue line) state (PBE0/6-311+G*/LanL2TZf). (d) Integrated transient absorption in the range of 7200–8200 cm^–1^ plotted vs time. The fitted curve is the convoluted single-exponential decay function with a Gaussian instrument response fwhm of 400 ns. The best-fit decay time constant (τ) is 1.5 μs. (e) Phosphorescence decay and a fitted decay time constant of 1.5 μs. (f) Schematic diagram of formation, decay, and transitions of triplet FIrpic. (g) MOs of T_6_–T_1_ and T_7_–T_1_ transitions.

Transitions from the optimized T_1_ state are calculated to have a vertical transition energy at 6859, 6938, 7432, 8085, and 10163 cm^–1^. The three low-energy bands have low oscillator strengths and thus are not considered. The other two bands correspond to the T_6_ ← T_1_ (137B ← 134B; *f* = 0.0328) and T_7_ ← T_1_ (143A ← 138A; *f* = 0.0114) transitions, as shown in Figure 4c. Accordingly, we assigned the 6200–8200 cm^–1^ band with large absorption to the T_6_ ← T_1_ transition and the band at 8600–10000 cm^–1^ to the T_7_ ← T_1_ transition. From the calculated MOs, as shown in Figure 4g, the T_7_ ← T_1_ transition involves intraligand-centered π′_dfppy_ ← π_dfppy_ transitions; therefore, it is a ^3^LC transition. The T_6_ ← T_1_ transition involved d′+π_dfppy_ ← d transitions; this is a ^3^MLCT ← ^3^d transition. On the basis of the calculation and the short lifetime, the T_1_ state should be a ^3^MLCT.

Tsai et al.^33^ and Lai et al.^34^ studied FIrpic and Ir(ppy)_3_ using Raman and infrared spectroscopy and found the agreement of the vibrational spectra with that calculated using DFT/B3LYP. This confirms the accuracy of the DFT ground state geometries and bonding strengths. Our calculations on TD-DFT show that, for Ir(ppy)_3_/Ir(piq)_3_, the T_1_ (*A* symmetry) state mostly resulted from the HOMO (metal-centered d orbital) → LUMO (cyclometalated ligand π*) transition, and T_2_ (*E* symmetry) is from the HOMO (d) → LUMO+1 (π*) transition; both lie close in energy (difference of ∼0.02 eV) and are quasi-degenerate MLCT states. They can be accessed via intersystem crossing from the singlet manifold.

For FIpic, using PBE0 the T_1_ state resulted from the HOMO (d) → LUMO+1 (dfppy π*) transition mixed with some contribution from the HOMO → LUMO transition (ancillary ligand picolinate π*) (these MOs are shown in the Supporting Information). B3LYP also yielded similar results. The T_2_ state (∼0.04 eV above the T_1_ state) is mostly from the HOMO → LUMO+2 transition (dfppy π*) and is also mixed with other transitions. Li et al. employed DFT/B3LYP and used in several heteroleptic iridium complexes and found that the ancillary ligand has a weak effect on the emission for FIrpic.^35^ States T_1_ and T_2_ can be accessed from relaxation of singlet states, and furthermore, the T_2_ state can internally convert into T_1_. With the nanosecond-sensitive photodiode, we cannot resolve this conversion process. The next two triplet states, T_3_ and T_4_, are also MLCTs with energies ∼0.41 eV above T_1_ (393 nm from S_0_), but they should not be involved in the triplet transient transition process, giving ultrafast internal conversion/ISC to lowest triplet states in iridium complex systems. Overall, for all three Ir complexes, only the vertical transitions based on the optimized T_1_ geometries were calculated, and these TD-DFT results provide excellent agreement with the experimental data.

The TD-DFT vertical transition energies and oscillator strengths from the T_1_ structures agree better with the experimentally observed band structures; this confirms that the DFT T_1_ geometries and their electronic structures are reliable. These data are used to assist in understanding the electronic characters of the triplet states. For RB, the intense triplet absorption band is reassigned to the T_3_ ← T_1_ transition, a π′ ← π transition. For the three Ir complexes, their lowest triplet states are assigned to ^3^MLCT, so they exhibit relatively short lifetimes and nice emission quantum yields. Their triplet transitions involving metal orbitals, for example, d or d+π to reach the high-lying triplet state ^3^MLCT, generally have greater oscillator strengths in the NIR region. The ancillary ligand picolinate in FIrpic has little effect on the transient triplet absorption spectra.

In the work presented here, only the absorption mode is employed, but these data show that the step-scan time-resolved FTNIR spectroscopic technique can provide an ingenious means of detecting triplet states or long-lived intermediates in a broad energy range. Because the window of detection also covers a broad time range, the reaction kinetics of those triplet species can be studied by using this technique in the future. The time-resolved FT spectrometer can be further extended to the mid-IR region, and some vibrational structures can be resolved in addition to the high-energy electronic states.^36^ However, absorption of the solvent becomes much stronger in this range, and this can hamper the detection sensitivity.

## Methods

Rose bengal (RB) (Alfa Aesar), Ir(ppy)_3_ (Nichem), Ir(piq)_3_ (Lumtec), and FIrpic (Shine Materials Technology) were used as received. The RB solution was prepared at 0.1 mM in methanol, acetonitrile, and THF; Ir(ppy)_3_ and FIrpic were prepared at ∼1 mM in THF, and Ir(piq)_3_ was at ∼570 μM in THF.

**Figure 5:**
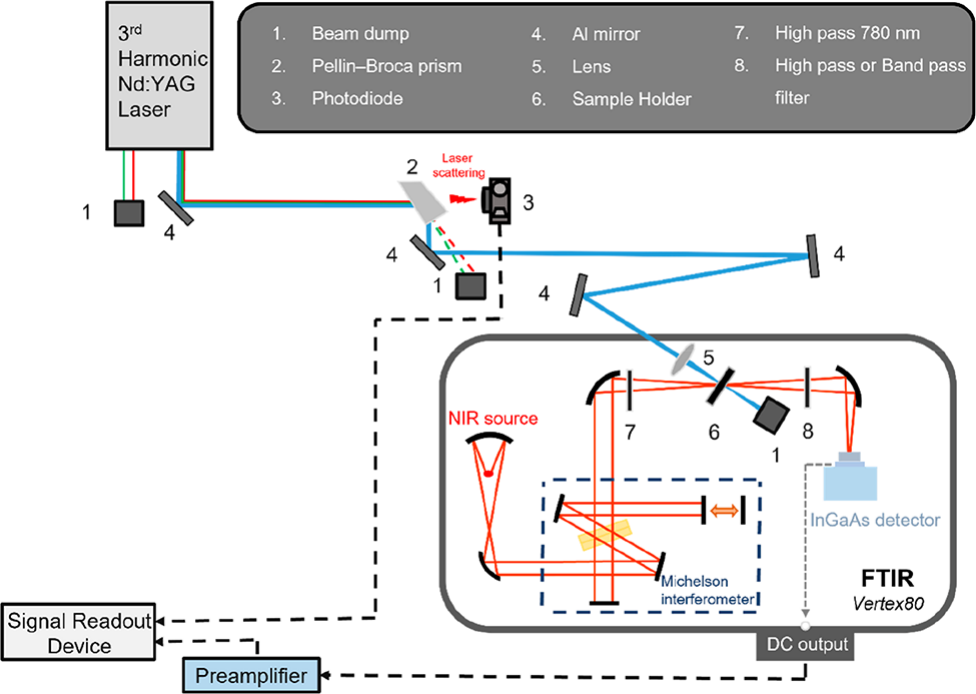
Schematic diagram of the experimental setup for the step-scan time-resolved FTNIR system. The setup included a laser excitation system, sample cell, step-scan time-resolved Michelson interferometer, InGaAs detector, and a data acquisition system.

A step-scan FT NIR spectrometer (Vertex80, Bruker) was used, and the setup is shown in Figure 5. A tungsten lamp inside the instrument served as the NIR light source. A Michelson interferometer was equipped with a CaF_2_ beam splitter. An indium gallium arsenide (InGaAs) detector was operated in the range of 6000–12000 cm^–1^. An ac/dc couple was used to acquire the difference spectra.^37,38^ The dc-coupled signal was used for the background and phase correction. Then, the detected near-infrared signal induced by the laser irradiation of the aforementioned samples was dc-coupled and sent to a preamplifier (SR560, Stanford Research Systems) for filtering and amplification prior to connecting to an analog-to-digital converter (ADC, 20 MHz, 14 bits, Spectrum Instrumentation). To prevent laser scatter from entering the instrument and interfering with the zero-crossing point of the internal He–Ne laser light, a high-pass filter (RG-780, LAMBDA) was placed in the front window of the sample compartment to block unwanted light scattering. Another high-pass filter (RG-830, LAMBDA) was installed in the back window of the sample compartment to prevent laser scatter and sample fluorescence from entering the detector. Depending on the specific experimental requirements, an additional high-pass filter (FL-009021, Semrock) and a bandpass filter (8400–2100 cm^–1^) may be used.

A Nd:YAG nanosecond laser (Quanta-Ray INDI-40) was operated at 10 Hz to generate the third harmonic at 355 nm to serve as the excitation light. The sample cell contained two quartz windows sandwiching a 2 mm thick and 10 mm inner diameter PTFE spacer to hold the sample solution. A small sample path length was used to limit the solvent absorption in the NIR region. The emission of the sample, either fluorescence or phosphorescence, was detected by a photodiode (DET410/M, Thorlabs), which had a response time of a few nanoseconds.

DFT calculations were carried out using GAUSSIAN16.^39^ The RB, Ir(ppy)_3_, and Ir(piq)_3_ geometries of the lowest singlet and triplet states were optimized using the B3LYP functional with the LanL2DZ basis sets for I and Ir atoms and the 6-31G* basis set for the other atoms. The FIrpic geometries were optimized using the PBE0 functional with the same basis set because this functional yielded the electronic transitions in better agreement with the measured UV–vis absorption. Both hybrid functional methods B3LYP and PBE0 predicted agreeable ground state geometries with the X-ray structure of FIrpic, which is in agreement with the results of Baranoff and Curchad.^28^

TD-DFT calculations were performed with the LanL2TZf basis sets for I and Ir atoms and the 6-311+G* basis set for the other atoms to achieve better accuracy in the vertical transition energies. All calculations were conducted considering the solvent effect that employed the polarizable continuum model.^40^ Because no spin–orbit interaction was included in the calculations, transitions to triplets from S_0_ yielded zero oscillator strengths. To obtain the oscillator strength for the triplet transitions, the TD-DFT calculation also used the optimized T_1_ structure. Because of differences in the optimized geometries of the S_0_ and T_1_ states, the energy differences from T_1_ to T_*n*_ were varied.

## Supporting Information Available

TCSPC experimental details, sample cell assembly, setup of the visible light emission detection, and DFT results of the calculated molecular orbitals and transition energies (PDF)
