# Supplementary material for: Investigation of Electronic Structures of Triplet States Using Step-Scan Time-Resolved Fourier-Transform Near-Infrared Spectroscopy
Source: J Phys Chem Lett. 2024 Jan 19;15(4):912–8. doi: 10.1021/acs.jpclett.3c03521 (PMC10839901; doi:10.1021/acs.jpclett.3c03521)
Supplement: Supplementary file 1 — jz3c03521_si_001.pdf [file jz3c03521_si_001.pdf]

Investigation of Electronic Structures of Triplet States Using Step-Scan Time-Resolved Fourier Transform Near-Infrared Spectroscopy

Chia Chun Wu, Yu-Xiang Tsai, Li-Kang Chu and I-Chia Chen**

Department of Chemistry, National Tsing Hua University, Hsinchu, Taiwan 300044, Republic of China

Supporting Information: TCSPC experimental details, sample cell assembly, setup of the visible light emission detection, and DFT results of the calculated molecular orbitals and the transition energies.

Experimental description of TCSPC

Captions of Figures and Tables

Figure S1. Sample cell assembly.

Figure S2. Experimental setup for time-resolved visible light emission detection.

Figure S3. (Left) Transient absorption of 10^{-4} M Rose Bengal in THF, acetonitrile (ACN), and methanol excited at 355 nm and (right) the integrated transient absorption at 9000 – 10500 cm^{-1} plotted versus time in different solvents and the fitted time constants.

Figure S4. Electronic configurations of RB S_0, T_1, T_2, and T_3 states.

Figure S5. Molecular orbital diagrams obtained for the optimized structure of RB T_1 state, where A and B represent α and β spin electron orbital, respectively.

Figure S6. Molecular orbital diagrams obtained for the optimized structure of $Ir(ppy)_3$ S_0 state. Method: B3LYP/6-311+G*/LanL2TZf.

Figure S7. Molecular orbitals of electronic transition T_1 to T_n state of $Ir(ppy)_3$ calculated using B3LYP/6-311+G*/LanL2TZf: (a) $T_6 \leftarrow T_1$, (b) $T_7 \leftarrow T_1$, (c) $T_8 \leftarrow T_1$, and (d) $T_9 \leftarrow T_1$. All hydrogen atoms are neglected, and A and B represent α and β spin electron orbital, respectively.

Figure S8. Change in lifetime under continuous laser irradiation of $Ir(ppy)_3T_1$ state in THF solution: (a) immediately after laser irradiation and (b) after 15 min of laser irradiation. THF was reacted with O_2 in solution to reduce quenching in $Ir(ppy)_3T_1$ state.

Figure S9. Molecular orbitals of electronic transition T_1 to T_n state of $Ir(piq)_3$ calculated using B3LYP/6-311+G*/LanL2TZf: (a) $T_5 \leftarrow T_1$, (b) $T_6 \leftarrow T_1$. All hydrogen atoms are neglected, and A and B represent α and β spin electron orbital, respectively.

Figure S10. Molecular orbital diagrams obtained for the optimized structure of $Ir(piq)_3$ S_0 state. Method: B3LYP/6-311+G*/LanL2TZf.

Figure S11. Molecular orbital diagrams obtained for the optimized structure of FIrpic S_0 state. Method: PBE0/6-311+G*/LanL2TZf.

Figure S12. Molecular orbitals of electronic transition T_1 to T_n state of FIrpic calculated using PBE0/6-311+G*/LanL2TZf: (a) $T_5 \leftarrow T_1$, (b) $T_6 \leftarrow T_1$. (c) $T_7 \leftarrow T_1$. All hydrogen atoms are neglected, and A and B represent α and β spin electron orbital, respectively.

Table S1. Vertical transitions, energies, and oscillator strengths of RB from the S_0 state calculated using B3LYP/6-311+G*/LanL2DZ.

Table S2. Vertical transitions, energies, and oscillator strengths from the optimized T_1 of RB to the T_n states calculated using B3LYP/6-311+G*/LanL2DZ.

Table S3. Vertical transitions, energies, and oscillator strengths of $Ir(ppy)_3$ from the S_0 state calculated using B3LYP/6-311+G*/LanL2TZf.

Table S4. Vertical transition energies from the optimized S_0 structure of $Ir(ppy)_3$ to the T_n states, and the energy differences between T_n and T_1, calculated using B3LYP/6-311+G*/LanL2TZf.

Table S5. Vertical transitions, energies, and oscillator strengths from the optimized T_1 structure of $Ir(ppy)_3$ to the T_n states calculated using B3LYP/6-311+G*/LanL2TZf.

Table S6. Vertical transitions, energies, and oscillator strengths of $Ir(piq)_3$ from the S_0 state calculated using B3LYP/6-311+G*/LanL2TZf.

Table S7. Vertical transition energies from the optimized S_0 structure of $Ir(piq)_3$ to the T_n states, and the energy differences between T_n and T_1, calculated using B3LYP/6-311+G*/LanL2TZf.

Table S8. Vertical transitions, energies, and oscillator strengths from the optimized T_1 structure of $Ir(piq)_3$ to the T_n states calculated using B3LYP/6-311+G*/LanL2TZf.

Table S9. Vertical transitions, energies, and oscillator strengths of FIrpic from the S_0 state calculated using PBE0/6-311+G*/LanL2TZf.

Table S10. Vertical transition energies from the optimized S_0 structure of FIrpic to the T_n states, and the energy differences between T_n and T_1, calculated using PBE0/6-311+G*/LanL2TZf.

Table S11. Vertical transitions, energies, and oscillator strengths from the optimized T_1 structure of FIrpic to the T_n states calculated using PBE0/6-311+G*/LanL2TZf.

Experimental description for time-correlated single-photon counting (TCSPC):

The fluorescence emission curves of RB were measured using the time-correlated single photon counting (TCSPC) technique. The samples were excited by the second harmonic output (515 nm) of a femtosecond Nd:Yb fiber laser (wavelength 1030 nm, 75 fs, 1.8 W, and 35.9 MHz. EKSPLA FF1000) with a type I BBO crystals. The scattering light was detected by a fast photodiode (TDA-200) to serve as the stop pulse in the measurements. The fluorescence emission was collected perpendicularly, passing through bandpass filters, and then detected by an MCP-PMT (Hamamatsu, R3809U-50). This signal was sent to the TCSPC board (PicoQuant, HydraHarp 400) to serve as the start pulse to obtain the emission curves. The polarization of the excitation laser pulses was set at the magic angle (54.7°) by a half-waveplate to avoid the variation of emission intensity due to molecular reorientation motion. The full width at half maximum (FWHM) of the emission curve from laser light scattering was about 38 ps. This corresponded a time response <20 ps which mainly resulted from temporal uncertainties of detectors and electronics.

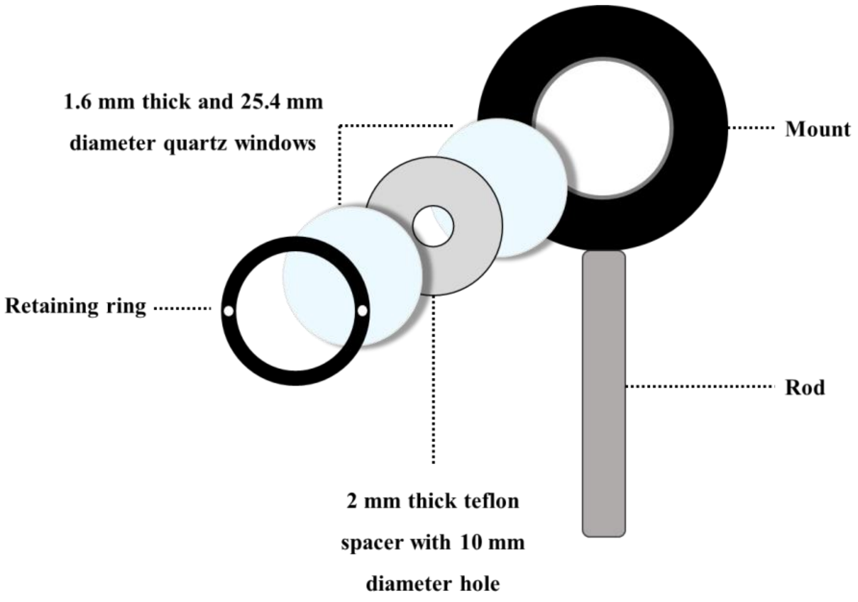

Figure S1. Sample cell assembly.

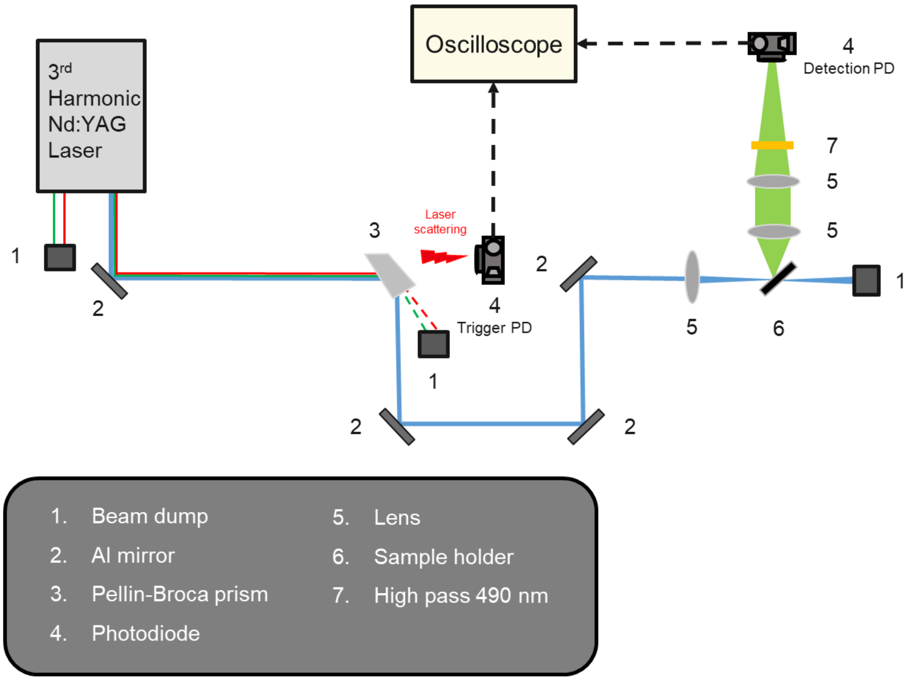

Figure S2. Experimental setup for time-resolved visible light emission detection.

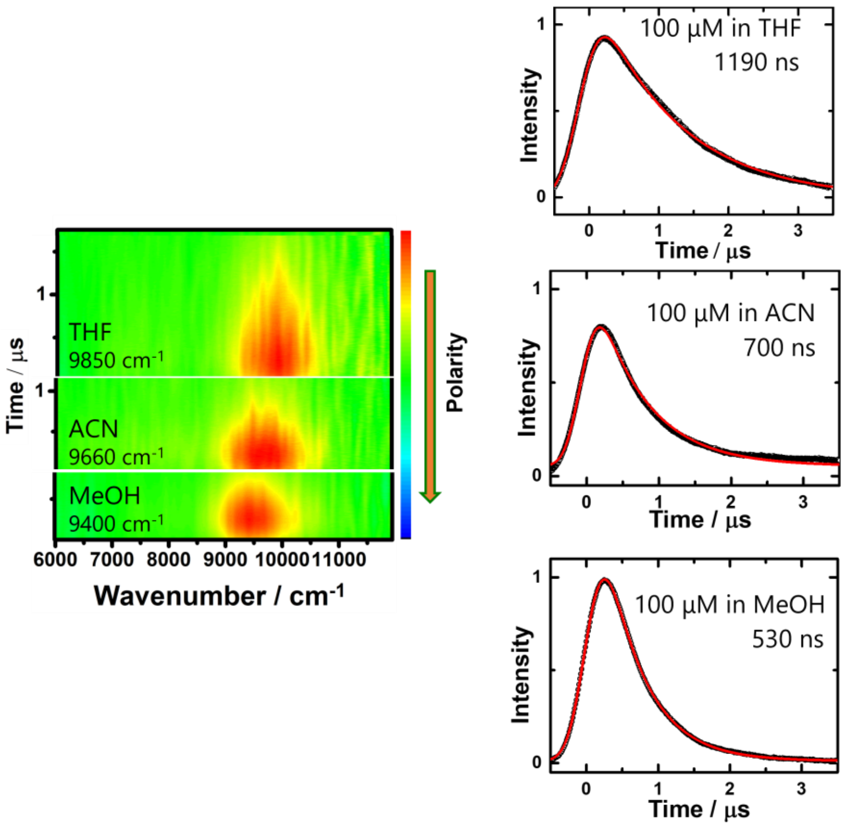

Figure S3. (Left) Transient absorption of 10^{-4} M Rose Bengal in THF, acetonitrile (ACN), and methanol excited at 355 nm and (right) the integrated transient absorption at 9000 – 10500 cm^{-1} plotted versus time in different solvents and the fitted time constants.

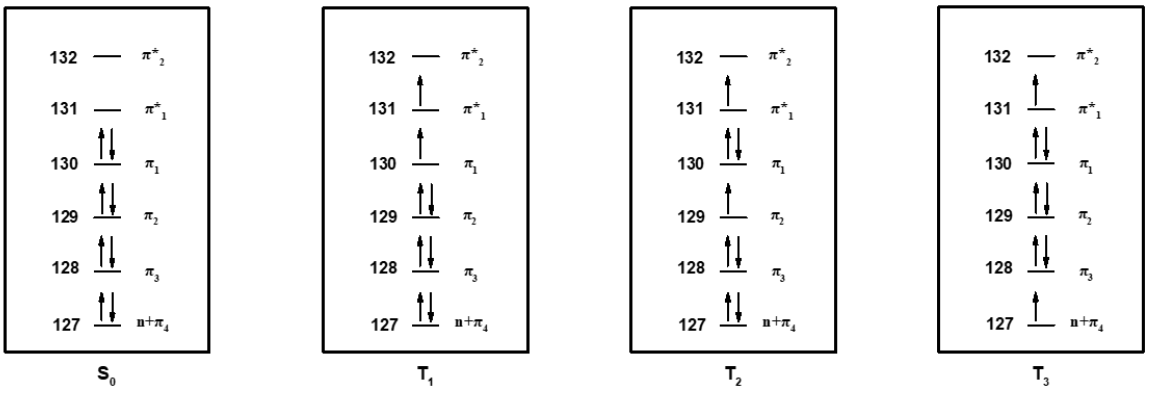

Figure S4. Electronic configurations of RB S_0, T_1, T_2, and T_3 states.

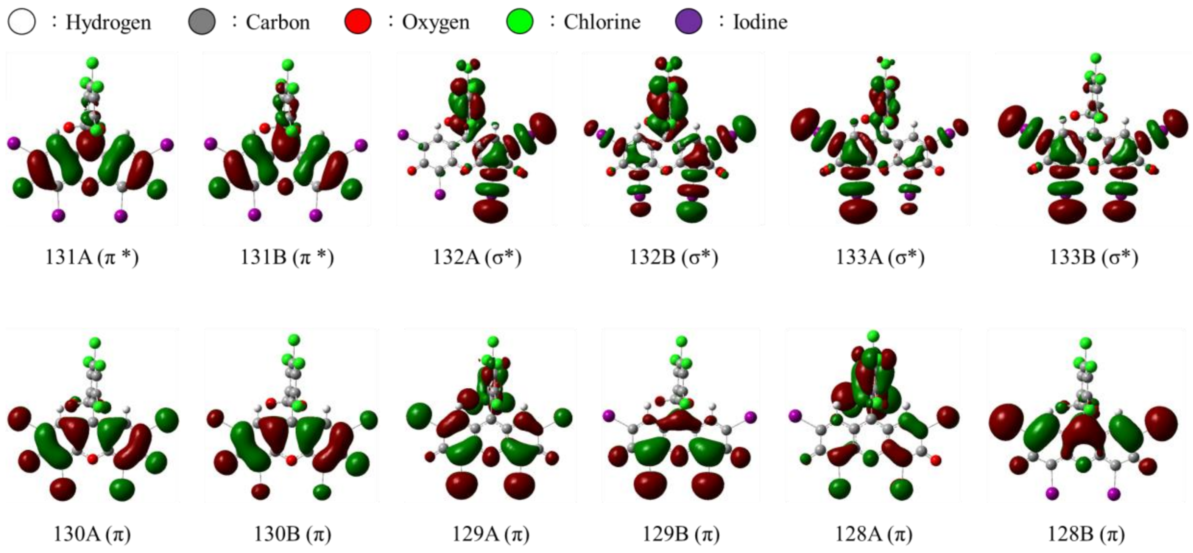

Figure S5. Molecular orbital diagrams obtained for the optimized structure of RB T_1 state, where A and B represent α and β spin electron orbital, respectively. Method: B3LYP/6-311+G*/LanL2TZf.

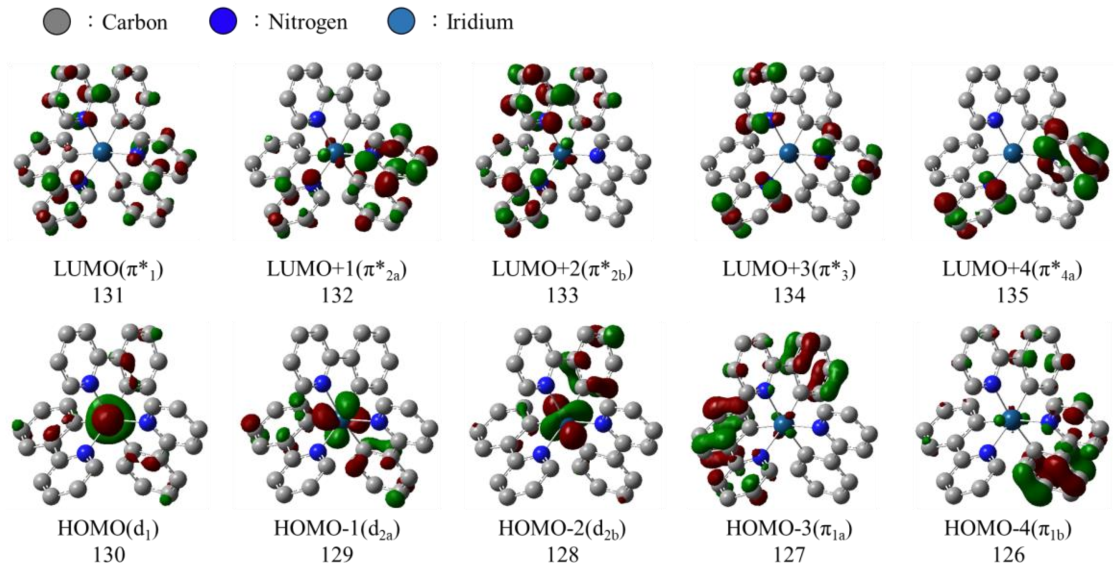

Figure S6. Molecular orbital diagrams obtained for the optimized structure of $Ir(ppy)_3$ S_0 state. Method: B3LYP/6-311+G*/LanL2TZf.

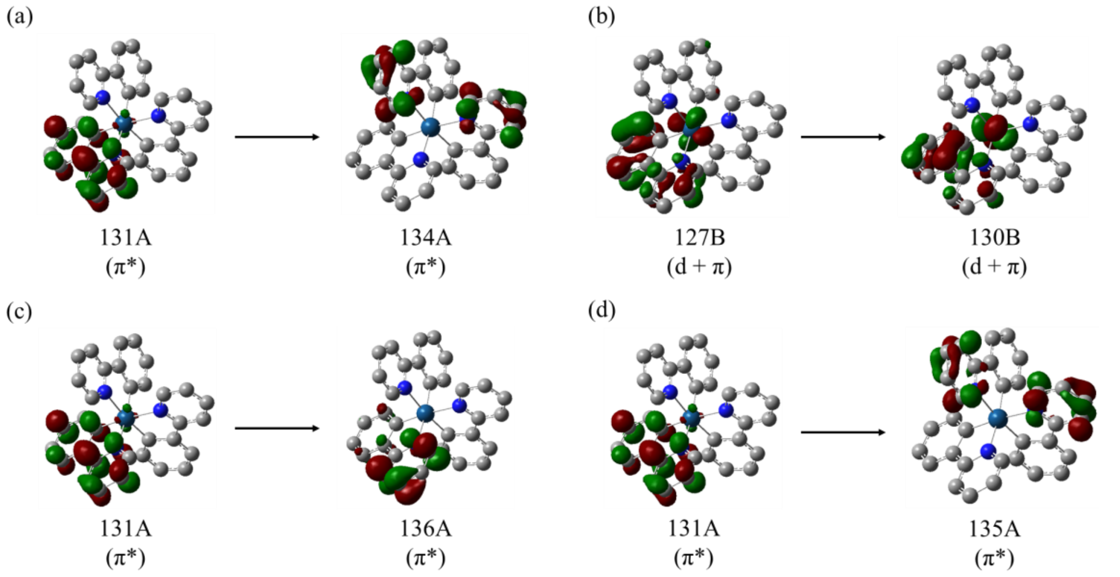

Figure S7. Molecular orbitals of electronic transition T_1 to T_n state of Ir(ppy)$_3$ calculated using B3LYP/6-311+G*/LanL2TZf: (a) $T_6 \leftarrow T_1$, (b) $T_7 \leftarrow T_1$, (c) $T_8 \leftarrow T_1$, and (d) $T_9 \leftarrow T_1$. All hydrogen atoms are neglected, and A and B represent α and β spin electron orbital, respectively.

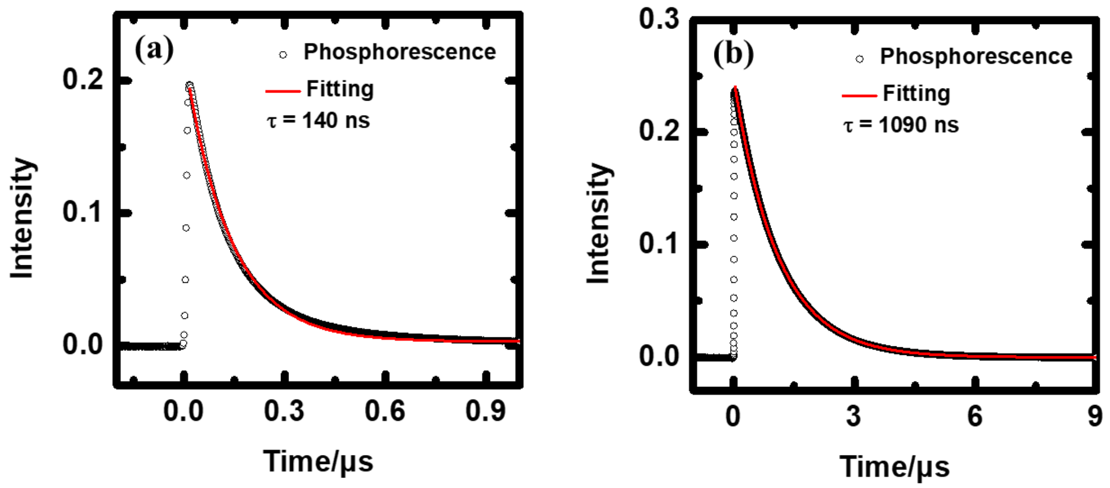

Figure S8. Change in lifetime under continuous laser irradiation of Ir(ppy)$_3$ T_1 state in THF solution: (a) immediately after laser irradiation and (b) after 15 min of laser irradiation. THF was reacted with O_2 in solution to reduce quenching in Ir(ppy)$_3$ T_1 state.

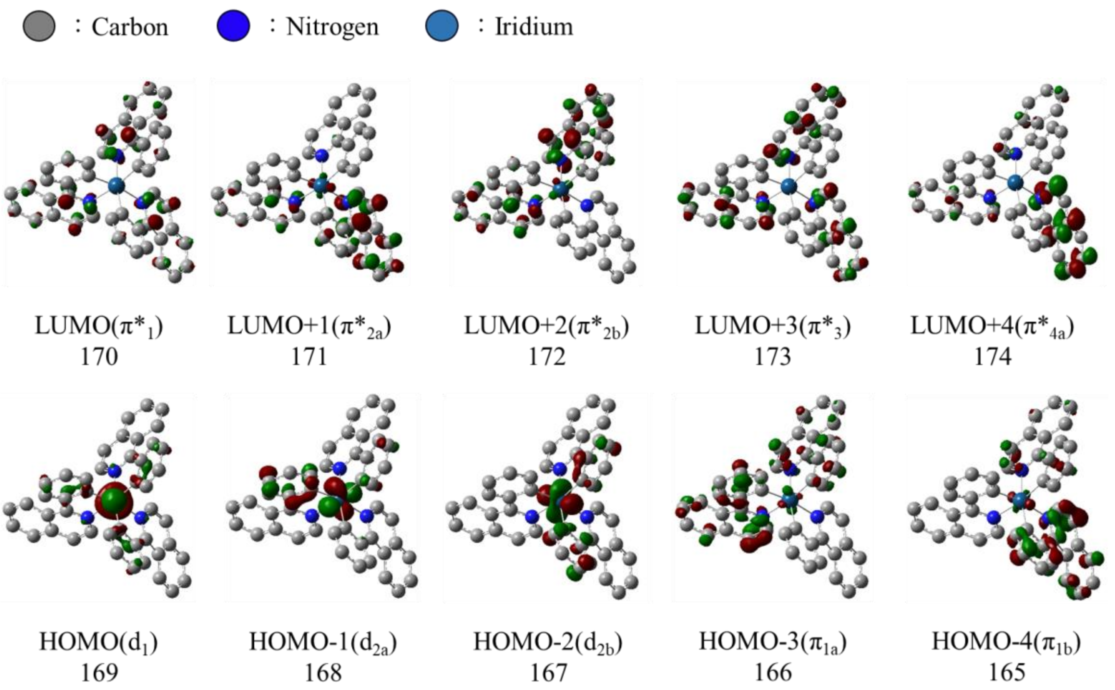

Figure S9. Molecular orbital diagrams obtained for the optimized structure of Ir(piq)$_3$ S_0 state. Method: B3LYP/6-311+G*/LanL2TZf.

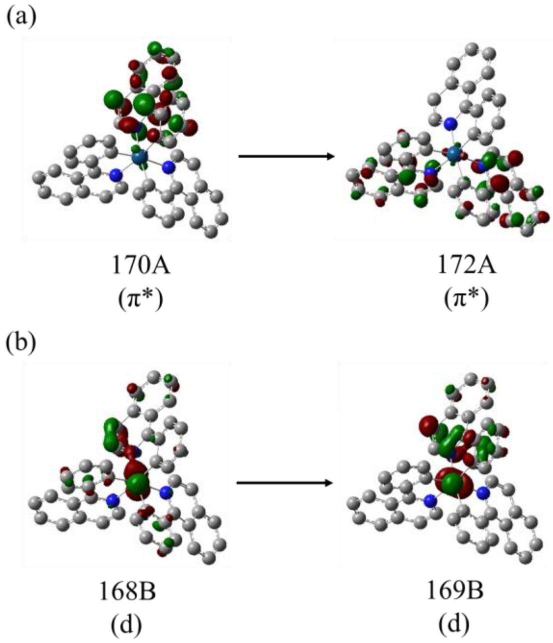

Figure S10. Molecular orbitals of electronic transition T_1 to T_n state of Ir(piq)$_3$ calculated using B3LYP/6-311+G*/LanL2TZf: (a) $T_5 \leftarrow T_1$, (b) $T_6 \leftarrow T_1$. All hydrogen atoms are neglected, and A and B represent α and β spin electron orbital, respectively.

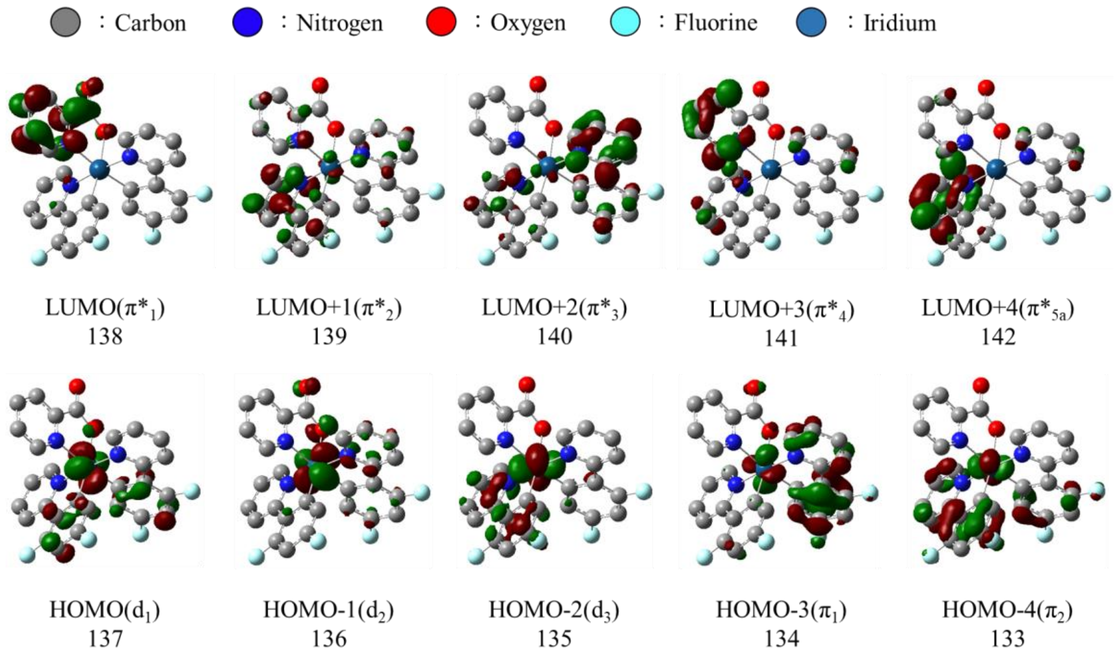

Figure S11. Molecular orbital diagrams obtained for the optimized structure of FIrpic S_0 state. Method: PBE0/6-311+G*/LanL2TZf.

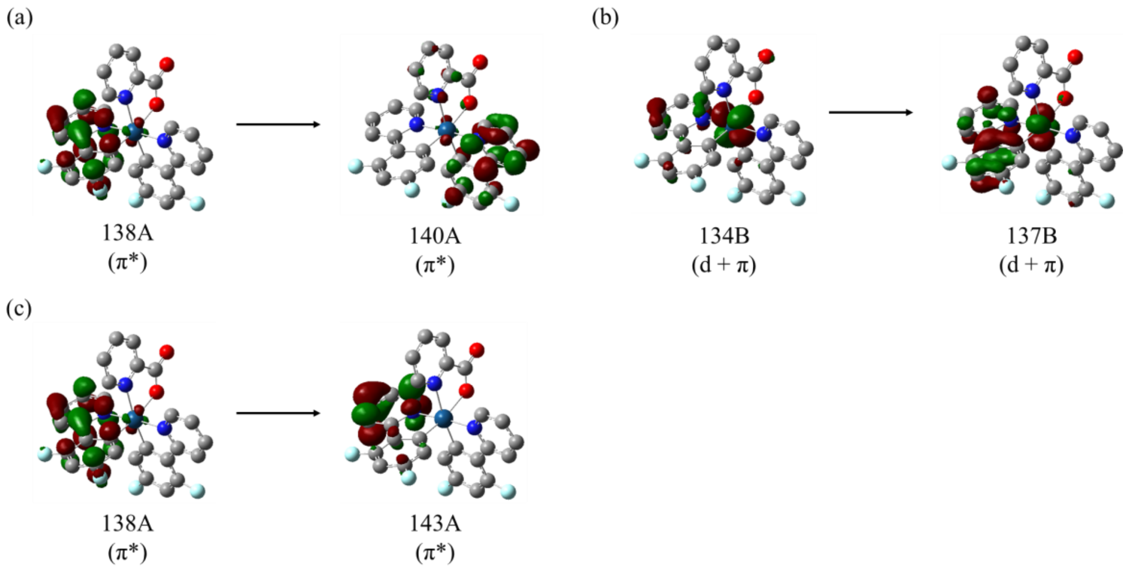

Figure S12. Molecular orbitals of electronic transition T_1 to T_n state of FIrpic calculated using PBE0/6-311+G*/LanL2TZf: (a) $T_5 \leftarrow T_1$, (b) $T_6 \leftarrow T_1$. (c) $T_7 \leftarrow T_1$. All hydrogen atoms are neglected, and A and B represent α and β spin electron orbital, respectively.

Table S1. Vertical transitions, energies, and oscillator strengths of RB from the S_0 state calculated using B3LYP/6-311+G*/LanL2DZ.

Singlets						Triplets				
Upper state	Dominant excitation	Coeff.	Energy (eV)	Wavelength (nm)	Oscillator strength	Upper state	Dominant excitation	Coeff.	Energy (eV)	Wavelength (nm)
S_1	130→131	0.70093	2.5921	478.31	0.7860	T_1	130→131	0.70545	2.5290	762.84
S_2	129→131	0.70165	2.9613	418.68	0.0016	T_2	129→131	0.68328	2.5290	490.26
S_3	128→131	0.70212	3.1626	392.03	0.0126	T_3	113→131	-0.15204	2.8150	440.44
S_4	118→131	-0.10445	3.3477	370.36	0.0003		114→131	-0.13807		
	122→131	0.10647					125→131	0.27944		
	126→131	0.63109					126→131	-0.18101		
	127→131	0.25394					127→131	0.55170		
S_5	130→132	0.51350	3.4019	364.46	0.0045	T_4	125→131	0.31882	3.0982	400.18
	130→133	-0.42374					126→131	0.12288		
	130→135	-0.13431					127→131	-0.10821		
	130→136	-0.15229					128→131	0.58329		
S_6	130→132	0.40786	3.4196	362.56	0.0032	T_5	118→131	-0.12941	3.1827	389.56
	130→133	0.53746					122→131	0.14743		
	130→134	0.10848					125→131	-0.35154		
	130→136	-0.12964					126→131	0.36845		
S_7	117→131	0.10241	3.4807	356.21	0.0123		127→131	0.32032		
	123→131	0.55857					128→131	0.17847		
	124→131	-0.12479					130→133	-0.14414		
	125→131	0.30947				T_6	126→131	0.10406	3.1994	387.52
	126→131	0.11013					130→132	-0.17201		
	127→131	-0.20190					130→133	0.59875		
S_8	123→131	-0.39465	3.4846	355.81	0.0244		130→135	0.11251		
	124→131	-0.20212				T_7	118→131	-0.10759	3.2036	387.01
	125→131	0.44177					125→131	0.34247		
	126→131	0.12317					126→131	0.46185		
	127→131	-0.28096					128→131	-0.32323		

Table S2. Vertical transitions, energies, and oscillator strengths from the optimized T_1 of RB to the T_n states calculated using B3LYP/6-311+G*/LanL2DZ.

Triplets					
Upper state	Dominant excitation	Coeff.	Energy (eV)	Wavelength (nm)	Oscillator strength
T_2	128B →130B	-0.34518	0.9190	1349.12	0.0202
	129B →130B	0.92481			
T_3	131A →140A	0.19482	1.326	935.01	0.2522
	128B →130B	0.91466			
	129B →130B	0.33105			
T_4	124B →130B	-0.19435	1.412	878.06	0.0009
	126B →130B	0.18869			
	127B →130B	0.95274			
T_5	117B →130B	-0.14614	1.4543	852.51	0.0005
	118B →130B	-0.19437			
	120B →130B	0.24361			
	123B →131B	0.13548			
	124B →130B	0.88129			
	127B →130B	0.20507			
T_6	115B →130B	0.28457	1.5643	792.58	0.0001
	119B →130B	0.13337			
	123B →130B	0.91317			
	124B →131B	0.13692			
	126B →130B	-0.11195			
T_7	123B →130B	0.11382	1.5831	783.19	0.019
	126B →130B	0.95626			
	127B →130B	-0.20521			
T_8	125B →130B	0.99443	1.784	694.98	0.0006
T_9	131A →132A	0.93093	1.7969	689.99	0.0035
	131A →133A	-0.33872			

Table S3. Vertical transitions, energies, and oscillator strengths of $Ir(ppy)_3$ from the S_0 state calculated using B3LYP/6-311+G*/LanL2TZf.

Singlets						Triplets				
Upper state	Dominant excitation	Coeff.	Energy (eV)	Wavelength (nm)	Oscillator strength	Upper state	Dominant excitation	Coeff.	Energy (eV)	Wavelength (nm)
S_1(A)	130→131	0.69786	2.8316	437.86	0.0095	T_1(A)	126→132	-0.11317	2.6026	476.39
S_2(E)	130→132	0.69339	2.9088	426.23	0.0045		127→133	0.11203		
	130→133	0.69349	2.9098	426.09	0.0046		128→133	0.20454		
S_3(E)	129→131	0.67822	3.0427	407.48	0.0499		129→132	-0.20703		
	128→131	0.67803	3.0438	407.33	0.0499		130→131	0.59202		
S_4(A)	128→132	0.27671	3.0898	401.27	0.0051	T_2(E)	126→131	-0.13906	2.629	471.6
	128→133	-0.40054					128→133	0.15447		
	129→132	0.40996					129→131	-0.28267		
	129→133	0.27745					129→132	0.15161		
S_5(E)	128→131	-0.12031	3.1584	392.55	0.0631		130→132	0.5368		
	128→132	0.48					127→131	0.13898	2.6296	471.49
	129→133	-0.47823					128→131	0.28169		
	128→133	0.48216	3.1585	392.54	0.0634		128→132	0.154		
	129→131	0.11924					129→133	-0.15357		
	129→132	0.47611					130→133	0.53711		
S_6(A)	0.25449	0.25449	3.1996	387.5	0	T_3(A)	128→132	0.36021	2.8526	434.64
	0.12761	0.12761					128→133	0.28456		
	-0.12023	-0.12023					129→132	-0.30289		
	0.25494	0.25494					129→133	0.35279		
	0.57271	0.57271					130→131	-0.17092		
S_7(A)	128→132	-0.31651	3.3606	368.93	0.0853	T_4(E)	128→131	-0.23344	2.8744	431.35
	128→133	-0.24128					128→132	0.28144		
	129→132	0.23931					129→131	0.42515		
	129→133	-0.31608					129→133	-0.29225		
	130→134	0.39211					130→132	0.25559		
S_8(E)	129→134	0.69354	3.4	364.66	0.0072		128→131	0.4237	2.8753	431.2
	128→134	0.69347	3.4011	364.54	0.0071		128→133	0.29644		

Table S2. Vertical transitions, energies, and oscillator strengths from the optimized T_1 of RB to the T_n states calculated using B3LYP/6-311+G*/LanL2DZ.

Triplets					
Upper state	Dominant excitation	Coeff.	Energy (eV)	Wavelength (nm)	Oscillator strength
T_2	128B →130B	-0.34518	0.9190	1349.12	0.0202
	129B →130B	0.92481			
T_3	131A →140A	0.19482	1.326	935.01	0.2522
	128B →130B	0.91466			
	129B →130B	0.33105			
T_4	124B →130B	-0.19435	1.412	878.06	0.0009
	126B →130B	0.18869			
	127B →130B	0.95274			
T_5	117B →130B	-0.14614	1.4543	852.51	0.0005
	118B →130B	-0.19437			
	120B →130B	0.24361			
	123B →131B	0.13548			
	124B →130B	0.88129			
	127B →130B	0.20507			
T_6	115B →130B	0.28457	1.5643	792.58	0.0001
	119B →130B	0.13337			
	123B →130B	0.91317			
	124B →131B	0.13692			
	126B →130B	-0.11195			
T_7	123B →130B	0.11382	1.5831	783.19	0.019
	126B →130B	0.95626			
	127B →130B	-0.20521			
T_8	125B →130B	0.99443	1.784	694.98	0.0006
T_9	131A →132A	0.93093	1.7969	689.99	0.0035
	131A →133A	-0.33872			

Table S3. Vertical transitions, energies, and oscillator strengths of Ir(ppy)$_3$ from the S_0 state calculated using B3LYP/6-311+G*/LanL2TZf. (Cont.)

Singlets						Triplets				
Upper state	Dominant excitation	Coeff.	Energy (eV)	Wavelength (nm)	Oscillator strength	Upper state	Dominant excitation	Coeff.	Energy (eV)	Wavelength (nm)
S_9(E)	130→135	0.68483	3.5282	351.41	0.0395		129→131	0.23457		
	130→136	0.68478	3.5288	351.35	0.0045		129→132	0.28061		
S_{10}(A)	128→136	-0.43417	3.6844	336.51	0.0014		130→133	-0.25179		
	129→135	0.55114				T_5(E)	128→131	0.22256	2.9883	414.9
S_{11}(E)	128→135	-0.48765	3.6926	335.77	0.012		128→132	-0.21615		
	129→136	0.49427					128→133	-0.25773		
	128→136	0.54587	3.6927	335.76	0.0119		129→131	0.29317		
	129→135	0.42921					129→132	-0.26304		
S_{12}(A)	128→135	0.48746	3.7201	333.28	0.0175		129→133	0.20469		
	129→136	0.48067					130→132	0.3057		
S_{13}(E)	127→131	0.67962	4.144	299.19	0.0236		128→131	-0.29562	2.9886	414.86
	126→131	0.67956	4.1444	299.16	0.0238		128→132	-0.25858		
S_{14}(A)	125→131	-0.22507	4.2346	292.79	0.0173		128→133	0.2053		
	126→132	0.39304					129→131	0.2213		
	126→133	-0.25307					129→132	0.21094		
	127→132	-0.28229					129→133	0.26143		
	127→133	-0.37149					130→133	0.30818		
S_{15}(E)	126→132	0.41716	4.2428	292.23	0.0822	T_6(A)	125→131	-0.10453	3.0243	409.96
	126→133	-0.21455					128→132	-0.2957		
	126→134	-0.11736					128→133	0.31894		
	127→132	0.22915					129→132	-0.30704		
	127→133	0.41411					129→133	-0.29787		
	126→132	0.20203	4.2429	292.22	0.0826		130→131	-0.28454		
	126→133	0.41648				T_7(A)	128→136	-0.12103	3.1258	396.65
	127→132	-0.41376					129→135	0.12108		
	127→133	0.24108					130→134	0.66505		
	127→134	-0.11802				T_8(E)	125→135	-0.10971	3.212	386

Table S2. Vertical transitions, energies, and oscillator strengths from the optimized T_1 of RB to the T_n states calculated using B3LYP/6-311+G*/LanL2DZ.

Triplets					
Upper state	Dominant excitation	Coeff.	Energy (eV)	Wavelength (nm)	Oscillator strength
T_2	128B →130B	-0.34518	0.9190	1349.12	0.0202
	129B →130B	0.92481			
T_3	131A →140A	0.19482	1.326	935.01	0.2522
	128B →130B	0.91466			
	129B →130B	0.33105			
T_4	124B →130B	-0.19435	1.412	878.06	0.0009
	126B →130B	0.18869			
	127B →130B	0.95274			
T_5	117B →130B	-0.14614	1.4543	852.51	0.0005
	118B →130B	-0.19437			
	120B →130B	0.24361			
	123B →131B	0.13548			
	124B →130B	0.88129			
	127B →130B	0.20507			
T_6	115B →130B	0.28457	1.5643	792.58	0.0001
	119B →130B	0.13337			
	123B →130B	0.91317			
	124B →131B	0.13692			
	126B →130B	-0.11195			
T_7	123B →130B	0.11382	1.5831	783.19	0.019
	126B →130B	0.95626			
	127B →130B	-0.20521			
T_8	125B →130B	0.99443	1.784	694.98	0.0006
T_9	131A →132A	0.93093	1.7969	689.99	0.0035
	131A →133A	-0.33872			

Table S3. Vertical transitions, energies, and oscillator strengths of Ir(ppy)$_3$ from the S_0 state calculated using B3LYP/6-311+G*/LanL2TZf. (Cont.)

Singlets						Triplets				
Upper state	Dominant excitation	Coeff.	Energy (eV)	Wavelength (nm)	Oscillator strength	Upper state	Dominant excitation	Coeff.	Energy (eV)	Wavelength (nm)
S_{16}(A)	125→131	-0.11436	4.2645	290.73	0.1332		126→134	0.10118		
	126→132	0.24216					128→135	-0.12885		
	126→133	0.42461					129→134	0.53115		
	127→132	0.40081					129→136	0.12806		
	127→133	-0.24408					130→135	0.24756		
S_{17}(A)	125→131	0.63608	4.2768	289.9	0.3343		125→136	0.1104	3.2129	385.89
	126→132	0.14341					127→134	0.10115		
	127→133	-0.14564					128→134	0.52873		
S_{18}(E)	125→132	0.62844	4.3411	285.61	0.0111		128→136	-0.12717		
	126→134	0.21926					129→135	-0.12958		
	125→133	0.62755	4.3419	285.55	0.0109		130→136	-0.24953		
	127→134	-0.22107				T_9(E)	125→132	0.25777	3.2467	381.88
S_{19}(E)	125→132	-0.20795	4.4639	277.75	0.0377		126→131	0.34482		
	125→133	0.16397					126→132	-0.19861		
	126→134	0.30659					126→134	-0.10662		
	127→134	0.53283					127→133	-0.19839		
	125→132	-0.16274	4.464	277.74	0.0378		128→134	-0.13295		
	125→133	-0.20984					129→131	-0.13943		
	126→134	0.53301					129→134	0.22356		
	127→134	-0.3059					130→132	0.11791		
S_{20}(A)	124→131	0.44308	4.5182	274.41	0.0201		125→133	-0.25734	3.247	381.84
	125→134	0.23923					126→133	-0.19864		
	130→137	-0.25312					127→131	0.34372		
	130→138	0.35404					127→132	0.19982		
S_{21}(A)	125→134	0.57378	4.5688	271.37	0.0738		127→134	-0.10584		
	130→137	0.18144					128→131	-0.1394		
	130→138	-0.19945					128→134	0.22646		

Table S2. Vertical transitions, energies, and oscillator strengths from the optimized T_1 of RB to the T_n states calculated using B3LYP/6-311+G*/LanL2DZ.

Triplets					
Upper state	Dominant excitation	Coeff.	Energy (eV)	Wavelength (nm)	Oscillator strength
T_2	128B →130B	-0.34518	0.9190	1349.12	0.0202
	129B →130B	0.92481			
T_3	131A →140A	0.19482	1.326	935.01	0.2522
	128B →130B	0.91466			
	129B →130B	0.33105			
T_4	124B →130B	-0.19435	1.412	878.06	0.0009
	126B →130B	0.18869			
	127B →130B	0.95274			
T_5	117B →130B	-0.14614	1.4543	852.51	0.0005
	118B →130B	-0.19437			
	120B →130B	0.24361			
	123B →131B	0.13548			
	124B →130B	0.88129			
	127B →130B	0.20507			
T_6	115B →130B	0.28457	1.5643	792.58	0.0001
	119B →130B	0.13337			
	123B →130B	0.91317			
	124B →131B	0.13692			
	126B →130B	-0.11195			
T_7	123B →130B	0.11382	1.5831	783.19	0.019
	126B →130B	0.95626			
	127B →130B	-0.20521			
T_8	125B →130B	0.99443	1.784	694.98	0.0006
T_9	131A →132A	0.93093	1.7969	689.99	0.0035
	131A →133A	-0.33872			

Table S3. Vertical transitions, energies, and oscillator strengths of Ir(ppy)$_3$ from the S_0 state calculated using B3LYP/6-311+G*/LanL2TZf. (Cont.)

Singlets						Triplets				
Upper state	Dominant excitation	Coeff.	Energy (eV)	Wavelength (nm)	Oscillator strength	Upper state	Dominant excitation	Coeff.	Energy (eV)	Wavelength (nm)
S_{22}(A)	121→131	0.18778	4.5833	270.51	0		129→134	0.13119		
	124→131	0.46193					130→133	-0.11784		
	125→134	-0.1463				T_{10}(A)	125→131	0.32531	3.2742	378.67
	130→137	0.30072					125→134	-0.13899		
	130→138	-0.28239					126→132	0.28184		
S_{23}(A)	123→131	-0.23927	4.6128	268.78	0.0237		126→135	0.12018		
	124→132	-0.11043					127→133	-0.28241		
	124→133	0.50977					127→136	-0.12041		
	130→139	-0.1807					128→132	-0.14104		
	130→140	-0.19899					128→133	0.11778		
	130→141	-0.1574					129→132	-0.11724		
	130→142	-0.10845					129→133	-0.14189		
S_{24}(E)	122→131	-0.26857	4.6629	265.9	0.0328		130→131	0.16146		
	123→131	-0.11305				T_{11}(A)	128→135	0.20386	3.4342	361.02
	129→137	-0.33649					128→136	0.38627		
	129→138	0.45155					129→135	-0.36212		
	130→139	-0.16859					129→136	0.18837		
	130→141	-0.10462					130→134	0.16252		
	130→142	0.10814				T_{12}(E)	126→131	0.10635	3.4379	360.64
	122→131	0.11092	4.6634	265.86	0.0323		128→134	0.18556		
	123→131	-0.27853					128→136	0.10459		
	128→137	-0.33375					129→134	-0.15924		
	128→138	0.4484					129→135	0.18167		
	130→140	0.16132					130→135	0.51418		
	130→141	0.10672					130→136	0.25169		
	130→142	0.10401					127→131	-0.10631	3.4383	360.6
S_{25}(E)	121→132	-0.14096	4.678	265.04	0.0076		128→134	0.16106		

Table S2. Vertical transitions, energies, and oscillator strengths from the optimized T_1 of RB to the T_n states calculated using B3LYP/6-311+G*/LanL2DZ.

Triplets					
Upper state	Dominant excitation	Coeff.	Energy (eV)	Wavelength (nm)	Oscillator strength
T_2	128B →130B	-0.34518	0.9190	1349.12	0.0202
	129B →130B	0.92481			
T_3	131A →140A	0.19482	1.326	935.01	0.2522
	128B →130B	0.91466			
	129B →130B	0.33105			
T_4	124B →130B	-0.19435	1.412	878.06	0.0009
	126B →130B	0.18869			
	127B →130B	0.95274			
T_5	117B →130B	-0.14614	1.4543	852.51	0.0005
	118B →130B	-0.19437			
	120B →130B	0.24361			
	123B →131B	0.13548			
	124B →130B	0.88129			
	127B →130B	0.20507			
T_6	115B →130B	0.28457	1.5643	792.58	0.0001
	119B →130B	0.13337			
	123B →130B	0.91317			
	124B →131B	0.13692			
	126B →130B	-0.11195			
T_7	123B →130B	0.11382	1.5831	783.19	0.019
	126B →130B	0.95626			
	127B →130B	-0.20521			
T_8	125B →130B	0.99443	1.784	694.98	0.0006
T_9	131A →132A	0.93093	1.7969	689.99	0.0035
	131A →133A	-0.33872			

Table S3. Vertical transitions, energies, and oscillator strengths of $Ir(ppy)_3$ from the S_0 state calculated using B3LYP/6-311+G*/LanL2TZf. (Cont.)

Singlets

Upper state	Dominant excitation	Coeff.	Energy (eV)	Wavelength (nm)	Oscillator strength
S_{26}(A)	126→135	0.18978	4.6927	264.21	0.0073
	127→136	-0.19046			
	130→137	0.48665			
	130→138	0.38985			
S_{27}(A)	122→132	0.10621	4.7051	263.51	0.0684
	122→133	-0.13435			
	123→132	-0.13572			
	123→133	-0.10787			
	125→134	0.1984			
	126→135	0.38405			
	127→136	-0.38108			
	130→137	-0.22136			
	130→138	-0.16309			

Triplets

Upper state	Dominant excitation	Coeff.	Energy (eV)	Wavelength (nm)
	128→139	-0.17806		
	128→141	-0.1356		
	129→140	-0.18442		
	129→142	-0.14628		
	130→137	-0.13278		
	130→138	0.24247		
T_{16}(E)	125→132	0.14614	3.8579	321.38
	125→135	-0.19066		
	126→131	0.15791		
	126→132	-0.12672		
	126→134	0.25316		
	126→135	0.1102		
	127→131	0.10686		
	127→133	-0.13489		
	127→134	0.15628		
	127→136	0.11732		
	128→140	-0.11775		
	129→137	0.10852		
	129→138	-0.20579		
	129→139	-0.11874		
	130→140	0.16423		
	130→142	0.13344		
	125→133	-0.14575	3.8582	321.35
	125→136	0.19082		
	126→131	-0.10674		
	126→133	-0.13361		
	126→134	-0.1556		

Table S2. Vertical transitions, energies, and oscillator strengths from the optimized T_1 of RB to the T_n states calculated using B3LYP/6-311+G*/LanL2DZ.

Triplets					
Upper state	Dominant excitation	Coeff.	Energy (eV)	Wavelength (nm)	Oscillator strength
T_2	128B →130B	-0.34518	0.9190	1349.12	0.0202
	129B →130B	0.92481			
T_3	131A →140A	0.19482	1.326	935.01	0.2522
	128B →130B	0.91466			
	129B →130B	0.33105			
T_4	124B →130B	-0.19435	1.412	878.06	0.0009
	126B →130B	0.18869			
	127B →130B	0.95274			
T_5	117B →130B	-0.14614	1.4543	852.51	0.0005
	118B →130B	-0.19437			
	120B →130B	0.24361			
	123B →131B	0.13548			
	124B →130B	0.88129			
	127B →130B	0.20507			
T_6	115B →130B	0.28457	1.5643	792.58	0.0001
	119B →130B	0.13337			
	123B →130B	0.91317			
	124B →131B	0.13692			
	126B →130B	-0.11195			
T_7	123B →130B	0.11382	1.5831	783.19	0.019
	126B →130B	0.95626			
	127B →130B	-0.20521			
T_8	125B →130B	0.99443	1.784	694.98	0.0006
T_9	131A →132A	0.93093	1.7969	689.99	0.0035
	131A →133A	-0.33872			

Table S3. Vertical transitions, energies, and oscillator strengths of $Ir(ppy)_3$ from the S_0 state calculated using B3LYP/6-311+G*/LanL2TZf. (Cont.)

Singlets						Triplets				
Upper state	Dominant excitation	Coeff.	Energy (eV)	Wavelength (nm)	Oscillator strength	Upper state	Dominant excitation	Coeff.	Energy (eV)	Wavelength (nm)
							126→136	0.11751		
							127→131	0.15747		
							127→132	0.1284		
							127→134	0.25225		
							127→135	-0.11112		
							128→137	0.10862		
							128→138	-0.20576		
							128→139	0.12578		
							129→140	-0.11004		
							130→139	0.16813		
							130→141	0.1318		
						T_{17}(A)	116→134	0.11314	4.0046	309.6
							124→131	0.12943		
							124→134	0.12891		
							125→134	0.26864		
							126→135	-0.14435		
							126→136	-0.10025		
							127→135	-0.10153		
							127→136	0.14195		
							128→136	-0.17602		
							128→139	0.12202		
							129→135	0.17924		
							129→140	0.1244		
							130→137	0.12273		
							130→138	-0.20727		
							130→146	0.11278		

Table S4. Vertical transition energies from the optimized S_0 structure of $Ir(ppy)_3$ to the T_n states, and the energy differences between T_n and T_1, calculated using B3LYP/6-311+G*/LanL2TZf.

Upper state	Vertical transition energy T_n–S_0 (eV)	Energy of T_n–T_1 (cm^{-1})
T_1(A)	2.6026	-
T_2(E)	2.6290	242
T_3(A)	2.8526	2016
T_4(E)	2.8744	2178
T_5(E)	2.9883	3146
T_6(A)	3.0243	3388
T_7(A)	3.1258	4275
T_8(E)	3.2120	4920
T_9(E)	3.2467	5243
T_{10}(A)	3.2742	5404
T_{11}(A)	3.4342	6694
T_{12}(E)	3.4379	6775
T_{13}(E)	3.5981	8100
T_{14}(A)	3.6600	8550
T_{15}(A)	3.8541	10082
T_{16}(E)	3.8579	10163

Table S5. Vertical transitions, energies, and oscillator strengths from the optimized T_1 structure of $Ir(ppy)_3$ to the T_n states calculated using B3LYP/6-311+G*/LanL2TZf.

Triplets					
Upper state	Dominant excitation	Coeff.	Energy(eV)	Wavelength (nm)	Oscillator strength
T_2(A)	123B→130B	0.12858	0.4428	2799.94	0.0042
	127B→130B	0.2992			
	128B→130B	-0.21449			
	129B→130B	0.8997			
T_3(A)	122B→130B	-0.15416	0.553	2242.01	0.001
	126B→130B	0.11403			
	127B→130B	0.17643			
	128B→130B	0.93317			
	129B→130B	0.15555			
T_4(A)	131A→132A	0.97215	0.6162	2012.09	0.0022
	131A→133A	-0.19084			
T_5(A)	131A→132A	0.19019	0.6762	1833.43	0.0014
	131A→133A	0.97731			
T_6(A)	131A→134A	0.80606	0.9552	1297.93	0.0068
	131A→136A	-0.52365			
	127B→130B	-0.22885			
T_7(A)	131A→134A	0.30794	0.9925	1249.2	0.0307
	127B→130B	0.87052			
	128B→130B	-0.11935			
	129B→130B	-0.32129			
T_8(A)	131A→134A	0.43677	1.1931	1039.16	0.0087
	131A→135A	0.33497			
	131A→136A	0.78981			
	127B→130B	-0.18599			
	129B→130B	0.11788			

Table S6. Vertical transitions, energies, and oscillator strengths of Ir(piq)$_3$ from the S$_0$ state calculated using B3LYP/6-311+G*/LanL2TZf.

Singlets						Triplets				
Upper state	Dominant excitation	Coeff.	Energy (eV)	Wavelength (nm)	Oscillator strength	Upper state	Dominant excitation	Coeff.	Energy (eV)	Wavelength (nm)
S$_1$(A)	169→ 170	0.69977	2.3093	536.89	0.0128	T$_1$(A)	164→ 170	-0.11013	2.0701	598.94
S$_2$(E)	167→ 170	0.41988	2.499	496.14	0.0567		165→ 171	0.14917		
	168→ 170	0.35202					166→ 172	-0.14877		
	169→ 171	-0.39094					167→ 172	0.13828		
	169→ 172	-0.193					168→ 171	-0.13858		
	167→ 170	-0.35031	2.4991	496.11	0.0567		169→ 170	0.61422		
	168→ 170	0.42233				T$_2$(E)	164→ 171	-0.12164	2.1665	572.28
	169→ 171	0.19577					165→ 170	0.21856		
	169→ 172	-0.38849					165→ 171	0.13645		
S$_3$(E)	167→ 170	0.38433	2.5496	486.29	0.0438		166→ 172	0.13762		
	168→ 170	0.20859					167→ 170	-0.13495		
	169→ 171	0.52633					168→ 170	-0.3196		
	169→ 172	0.13944					169→ 171	0.4651		
	167→ 170	-0.21011	2.5499	486.24	0.0432		164→ 172	-0.12126	2.1667	572.22
	168→ 170	0.38092					165→ 172	-0.13762		
	169→ 171	-0.13764					166→ 170	-0.2181		
	169→ 172	0.52867					166→ 171	0.1378		
S$_4$(A)	167→ 172	-0.48912	2.7056	458.26	0.0117		167→ 170	0.31935		
	168→ 171	0.49724					168→ 170	-0.13457		
S$_5$(E)	167→ 171	0.41913	2.7486	451.08	0.0609		169→ 172	0.46524		
	167→ 172	-0.26275				T$_3$(E)	167→ 170	0.53869	2.4037	515.81
	168→ 171	-0.25899					167→ 171	0.2037		
	168→ 172	-0.41828					168→ 170	-0.15693		
	167→ 171	0.26093	2.7487	451.06	0.0613		168→ 172	-0.20204		
	167→ 172	0.42232					169→ 172	-0.24192		
	168→ 171	0.41503					167→ 170	0.15666	2.4039	515.77

Table S5. Vertical transitions, energies, and oscillator strengths from the optimized T_1 structure of $Ir(ppy)_3$ to the T_n states calculated using B3LYP/6-311+G*/LanL2TZf.

Triplets					
Upper state	Dominant excitation	Coeff.	Energy(eV)	Wavelength (nm)	Oscillator strength
T_2(A)	123B→130B	0.12858	0.4428	2799.94	0.0042
	127B→130B	0.2992			
	128B→130B	-0.21449			
	129B→130B	0.8997			
T_3(A)	122B→130B	-0.15416	0.553	2242.01	0.001
	126B→130B	0.11403			
	127B→130B	0.17643			
	128B→130B	0.93317			
	129B→130B	0.15555			
T_4(A)	131A→132A	0.97215	0.6162	2012.09	0.0022
	131A→133A	-0.19084			
T_5(A)	131A→132A	0.19019	0.6762	1833.43	0.0014
	131A→133A	0.97731			
T_6(A)	131A→134A	0.80606	0.9552	1297.93	0.0068
	131A→136A	-0.52365			
	127B→130B	-0.22885			
T_7(A)	131A→134A	0.30794	0.9925	1249.2	0.0307
	127B→130B	0.87052			
	128B→130B	-0.11935			
	129B→130B	-0.32129			
T_8(A)	131A→134A	0.43677	1.1931	1039.16	0.0087
	131A→135A	0.33497			
	131A→136A	0.78981			
	127B→130B	-0.18599			
	129B→130B	0.11788			

Table S6. Vertical transitions, energies, and oscillator strengths of $Ir(piq)_3$ from the S_0 state calculated using B3LYP/6-311+G*/LanL2TZf. (Cont.)

Singlets						Triplets				
Upper state	Dominant excitation	Coeff.	Energy (eV)	Wavelength (nm)	Oscillator strength	Upper state	Dominant excitation	Coeff.	Energy (eV)	Wavelength (nm)
	168→ 172	-0.26079					167→ 172	-0.2032		
S_6(A)	167→ 171	0.48878	2.8798	430.53	0.0536		168→ 170	0.53844		
	168→ 172	0.48962					168→ 171	-0.20336		
S_7(A)	164→ 170	-0.10541	3.3771	367.13	0.0069		169→ 171	0.24212		
	169→ 173	0.67911				T_4(A)	167→ 171	0.47505	2.4857	498.79
S_8(E)	166→ 170	0.68439	3.4033	364.31	0.0079		168→ 172	0.47558		
	165→ 170	0.68418	3.4036	364.27	0.0081	T_5(A)	164→ 170	0.20131	2.566	483.18
S_9(E)	167→ 173	0.19524	3.5013	354.11	0.0355		165→ 171	-0.16278		
	168→ 173	0.18723					166→ 172	0.15933		
	169→ 174	0.62902					167→ 172	-0.35277		
	167→ 173	-0.18801	3.5017	354.06	0.0355		168→ 171	0.38604		
	168→ 173	0.19631					169→ 170	0.31638		
	169→ 175	0.62849				T_6(E)	164→ 171	0.10024	2.5739	481.7
S_{10}(E)	165→ 172	-0.11405	3.548	349.45	0.0214		165→ 170	-0.10822		
	166→ 170	0.10355					167→ 172	0.36765		
	166→ 171	0.11576					168→ 170	0.14598		
	167→ 173	0.29103					168→ 171	0.33326		
	168→ 173	0.54149					168→ 172	-0.10311		
	169→ 174	-0.2539					169→ 171	0.39473		
	165→ 170	-0.10437	3.5482	349.43	0.0215		164→ 172	0.10031	2.5744	481.6
	165→ 171	0.11474					166→ 170	0.10809		
	166→ 172	0.11435					167→ 170	-0.14596		
	167→ 173	0.54133					167→ 171	0.35061		
	168→ 173	-0.29038					167→ 172	-0.1008		
	169→ 175	0.25518					168→ 171	-0.10014		
S_{11}(A)	164→ 170	0.59715	3.5802	346.31	0.2071		168→ 172	-0.35151		

Table S5. Vertical transitions, energies, and oscillator strengths from the optimized T_1 structure of $Ir(ppy)_3$ to the T_n states calculated using B3LYP/6-311+G*/LanL2TZf.

Triplets					
Upper state	Dominant excitation	Coeff.	Energy(eV)	Wavelength (nm)	Oscillator strength
T_2(A)	123B→130B	0.12858	0.4428	2799.94	0.0042
	127B→130B	0.2992			
	128B→130B	-0.21449			
	129B→130B	0.8997			
T_3(A)	122B→130B	-0.15416	0.553	2242.01	0.001
	126B→130B	0.11403			
	127B→130B	0.17643			
	128B→130B	0.93317			
	129B→130B	0.15555			
T_4(A)	131A→132A	0.97215	0.6162	2012.09	0.0022
	131A→133A	-0.19084			
T_5(A)	131A→132A	0.19019	0.6762	1833.43	0.0014
	131A→133A	0.97731			
T_6(A)	131A→134A	0.80606	0.9552	1297.93	0.0068
	131A→136A	-0.52365			
	127B→130B	-0.22885			
T_7(A)	131A→134A	0.30794	0.9925	1249.2	0.0307
	127B→130B	0.87052			
	128B→130B	-0.11935			
	129B→130B	-0.32129			
T_8(A)	131A→134A	0.43677	1.1931	1039.16	0.0087
	131A→135A	0.33497			
	131A→136A	0.78981			
	127B→130B	-0.18599			
	129B→130B	0.11788			

Table S6. Vertical transitions, energies, and oscillator strengths of $Ir(piq)_3$ from the S_0 state calculated using B3LYP/6-311+G*/LanL2TZf. (Cont.)

Singlets						Triplets				
Upper state	Dominant excitation	Coeff.	Energy (eV)	Wavelength (nm)	Oscillator strength	Upper state	Dominant excitation	Coeff.	Energy (eV)	Wavelength (nm)
	165→ 171	-0.10387					169→ 172	0.3949		
	166→ 172	0.10474				T_7(E)	164→ 171	-0.24109	2.7847	445.24
	167→ 174	0.17536					165→ 170	0.37255		
	167→ 175	0.13246					165→ 171	0.20392		
	168→ 174	-0.13437					166→ 172	0.20155		
	168→ 175	0.17517					167→ 172	0.21564		
	169→ 173	0.10473					168→ 170	0.17075		
S_{12}(A)	164→ 170	-0.2424	3.6198	342.51	0.0618		168→ 171	0.21884		
	165→ 171	-0.25037					169→ 171	-0.20827		
	165→ 172	0.34136					164→ 172	0.24036	2.785	445.18
	166→ 171	0.35693					165→ 172	0.20363		
	166→ 172	0.25007					166→ 170	0.3718		
	167→ 174	0.17174					166→ 171	-0.20392		
	168→ 175	0.17002					167→ 170	0.1704		
S_{13}(E)	165→ 172	0.45262	3.6354	341.05	0.0837		167→ 171	-0.21774		
	166→ 171	-0.45239					168→ 172	0.21691		
	167→ 175	-0.11913					169→ 172	0.20858		
	168→ 173	0.14769				T_8(A)	164→ 170	0.30511	2.8285	438.33
	168→ 174	-0.11998					165→ 171	-0.26378		
	165→ 171	0.4541	3.6355	341.04	0.0835		165→ 172	0.10458		
	166→ 172	0.45233					166→ 171	0.10428		
	167→ 173	-0.14747					166→ 172	0.26613		
	167→ 174	-0.11772					167→ 172	0.29569		
	168→ 175	0.11875					168→ 171	-0.29256		
							169→ 170	0.10308		

Table S7. Vertical transition energies from the optimized S_0 structure of $Ir(piq)_3$ to the T_n states, and the energy differences between T_n and T_1, calculated using B3LYP/6-311+G*/LanL2TZf.

Upper state	Vertical transition energy T_n–S_0 (eV)	Energy of T_n–T_1 (cm^{-1})
T_1(A)	2.0701	-
T_2(E)	2.1665	807
T_3(E)	2.4037	2662
T_4(A)	2.4857	3388
T_5(A)	2.5660	3952
T_6(E)	2.5739	4033
T_7(E)	2.7847	5807
T_8(A)	2.8285	6130

Table S8. Vertical transitions, energies, and oscillator strengths from the optimized T_1 structure of $Ir(piq)_3$ to the T_n states calculated using B3LYP/6-311+G*/LanL2TZf.

Triplets					
Upper state	Dominant excitation	Coeff.	Energy(eV)	Wavelength (nm)	Oscillator strength
T_2(A)	123B→130B	0.12858	0.4428	2799.94	0.0042
	127B→130B	0.2992			
	128B→130B	-0.21449			
	129B→130B	0.8997			
T_3(A)	122B→130B	-0.15416	0.553	2242.01	0.001
	126B→130B	0.11403			
	127B→130B	0.17643			
	128B→130B	0.93317			
	129B→130B	0.15555			
T_4(A)	131A→132A	0.97215	0.6162	2012.09	0.0022
	131A→133A	-0.19084			
T_5(A)	131A→132A	0.19019	0.6762	1833.43	0.0014
	131A→133A	0.97731			
T_6(A)	131A→134A	0.80606	0.9552	1297.93	0.0068
	131A→136A	-0.52365			
	127B→130B	-0.22885			
T_7(A)	131A→134A	0.30794	0.9925	1249.2	0.0307
	127B→130B	0.87052			
	128B→130B	-0.11935			
	129B→130B	-0.32129			
T_8(A)	131A→134A	0.43677	1.1931	1039.16	0.0087
	131A→135A	0.33497			
	131A→136A	0.78981			
	127B→130B	-0.18599			
	129B→130B	0.11788			

Table S9. Vertical transitions, energies, and oscillator strengths of FIrpic from the S_0 state calculated using PBE0/6-311+G*/LanL2TZf.

Singlets					
Upper state	Dominant excitation	Coeff.	Energy (eV)	Wavelength (nm)	Oscillator strength
S_1(A)	137→138	-0.25274	3.1717	390.91	0.0592
	137→139	0.64117			
S_2(A)	137→138	0.64056	3.2164	385.47	0.0035
	137→139	0.26308			
	137→140	-0.10508			
S_3(A)	137→138	0.12693	3.2966	376.1	0.0011
	137→140	0.67824			
S_4(A)	135→139	-0.15843	3.6607	338.69	0.005
	136→139	0.62145			
	136→140	-0.23976			
S_5(A)	134→138	-0.12649	3.6761	337.27	0.0341
	135→138	-0.13391			
	136→138	0.67421			
S_6(A)	136→140	0.1777	3.7728	328.63	0.0065
	137→141	0.6677			
S_7(A)	134→140	-0.1143	3.8163	324.88	0.0902
	135→138	-0.22186			
	136→139	0.20207			
	136→140	0.56254			
	137→141	-0.18784			
S_8(A)	133→138	-0.10984	3.871	320.29	0.0673
	133→139	0.11815			
	134→138	0.16467			
	134→139	-0.16589			
	135→138	-0.38436			

Triplets				
Upper state	Dominant excitation	Coeff.	Energy (eV)	Wavelength (nm)
T_1(A)	133→139	0.11622	2.7487	451.06
	134→140	0.16693		
	135→139	-0.15316		
	135→140	0.15706		
	136→140	0.22616		
	137→138	-0.23136		
	137→139	0.43403		
	137→140	0.12031		
	137→144	0.10365		
T_2(A)	133→140	0.11579	2.7888	444.58
	133→142	-0.1101		
	134→139	0.11461		
	134→140	0.12933		
	135→138	-0.11366		
	135→139	0.2493		
	136→138	-0.10211		
	136→139	0.18405		
	136→140	0.14989		
	137→139	-0.14264		
	137→140	0.41788		
T_3(A)	135→138	-0.11442	3.1559	392.87
	135→139	0.12135		
	136→140	-0.18923		
	137→138	0.33707		
	137→139	0.47037		

Table S9. Vertical transitions, energies, and oscillator strengths of FIrpic from the S_0 state calculated using PBE0/6-311+G*/LanL2TZf. (Cont.)

Singlets						Triplets				
Upper state	Dominant excitation	Coeff.	Energy (eV)	Wavelength (nm)	Oscillator strength	Upper state	Dominant excitation	Coeff.	Energy (eV)	Wavelength (nm)
	135→139	0.40727					137→140	0.12324		
	135→140	0.21093				T_4(A)	134→143	0.11133	3.1662	391.59
	136→140	-0.17357					136→139	-0.21207		
S_9(A)	133→138	0.11324	3.9205	316.24	0.0524		136→140	-0.29092		
	133→140	0.15585					137→138	-0.33903		
	134→138	-0.17701					137→140	0.39105		
	134→140	-0.16219				T_5(A)	134→143	0.11133	3.1662	391.59
	135→138	0.38892				T_6(A)	133→138	-0.1025	3.1963	387.9
	135→139	0.18728					133→142	0.12638		
	135→140	0.41523					134→139	0.1516		
	136→139	0.1076					135→139	-0.29742		
S_{10}(A)	133→140	0.11869	3.9939	310.43	0.0253		135→140	0.16667		
	134→139	0.1138					137→138	0.4008		
	134→140	-0.10239					137→139	-0.15387		
	135→138	-0.15443					137→140	0.25622		
	135→139	-0.32909				T_7(A)	123→138	-0.14739	3.3259	372.78
	135→140	0.34229					129→138	0.10727		
	137→142	0.38605					129→141	-0.10989		
	137→143	0.10805					130→138	0.16669		
S_{11}(A)	135→139	0.25186	4.0691	304.7	0.0147		130→141	-0.13449		
	135→140	-0.19656					133→138	0.11771		
	136→139	0.10285					133→139	0.11452		
	137→142	0.55518					134→138	-0.22547		
	137→143	-0.19811					134→139	-0.1245		
S_{12}(A)	134→140	0.12437	4.0736	304.36	0.0062		135→138	0.27515		

Table S9. Vertical transitions, energies, and oscillator strengths of FIrpic from the S_0 state calculated using PBE0/6-311+G*/LanL2TZf. (Cont.)

Singlets						Triplets				
Upper state	Dominant excitation	Coeff.	Energy (eV)	Wavelength (nm)	Oscillator strength	Upper state	Dominant excitation	Coeff.	Energy (eV)	Wavelength (nm)
	135→139	0.1575					136→138	0.27668		
	137→143	0.63193					136→139	0.14162		
S_{13}(A)	131→138	-0.16023	4.1665	297.57	0.0452		137→138	0.1665		
	134→138	0.36252				T_8(A)	133→139	-0.19231	3.4443	359.97
	134→139	-0.27948					133→140	-0.11408		
	134→140	-0.20394					134→139	-0.17148		
	135→138	0.19193					134→140	-0.28388		
	136→141	0.28466					135→139	-0.17622		
	136→143	-0.12331					135→140	-0.12312		
	137→143	0.13121					136→138	0.16455		
S_{14}(A)	131→138	0.3507	4.1935	295.66	0.0242		136→139	0.28988		
	131→139	0.14937					136→140	0.1116		
	132→138	0.17757					136→141	-0.10983		
	134→138	-0.26072					136→143	0.10759		
	134→139	-0.32887					137→140	0.13024		
	134→140	-0.15314				T_9(A)	128→138	-0.10535	3.4767	356.62
	135→138	-0.11006					133→138	-0.10655		
	135→139	-0.11694					133→140	0.13829		
	136→141	0.16125					134→138	-0.13934		
S_{15}(A)	133→138	-0.11491	4.2408	292.36	0.1047		134→139	0.10623		
	133→139	0.24683					134→140	0.15633		
	134→138	0.11202					135→138	-0.23521		
	134→139	-0.27199					135→140	0.11157		
	134→140	0.342					136→138	0.44163		
	135→141	0.19081					136→139	0.15271		

Table S9. Vertical transitions, energies, and oscillator strengths of FIrpic from the S_0 state calculated using PBE0/6-311+G*/LanL2TZf. (Cont.)

Singlets							Triplets				
Upper state	Dominant excitation	Coeff.	Energy (eV)	Wavelength (nm)	Oscillator strength		Upper state	Dominant excitation	Coeff.	Energy (eV)	Wavelength (nm)
	135→142	0.13308						136→140	-0.13116		
	136→141	-0.27951						137→140	-0.11064		
S_{16}(A)	131→138	-0.16528	4.2792	289.74	0.1025		T_{10}(A)	132→139	-0.11601	3.5158	352.65
	133→139	0.19744						132→144	0.10575		
	134→138	-0.22288						133→138	-0.16841		
	134→140	0.24666						133→139	0.30018		
	134→141	-0.14737						133→140	-0.18627		
	135→140	0.12275						134→139	-0.14426		
	136→141	0.45635						134→142	0.10324		
	136→143	0.10979						135→138	-0.10585		
S_{17}(A)	131→138	0.33234	4.3064	287.9	0.0251			137→139	-0.10255		
	131→139	0.14341						137→141	-0.2355		
	132→138	0.20958						137→143	0.10702		
	133→138	-0.24883						137→144	0.11959		
	134→138	0.22785					T_{11}(A)	133→139	0.12823	3.569	347.39
	134→139	0.30634						133→140	-0.12927		
	135→138	0.15185						134→139	-0.10023		
	136→141	0.18858						134→143	-0.13345		
S_{18}(A)	131→138	-0.11237	4.3349	286.01	0.0336			135→139	0.18425		
	133→138	-0.24296						135→142	-0.11908		
	133→139	0.36608						136→138	0.16122		
	134→138	-0.20836						136→139	-0.26028		
	134→139	0.14264						137→141	0.39632		
	134→140	-0.33794						137→143	-0.23368		
	135→140	-0.12987					T_{12}(A)	134→139	-0.13553	3.6208	342.43

Table S9. Vertical transitions, energies, and oscillator strengths of FIrpic from the S_0 state calculated using PBE0/6-311+G*/LanL2TZf. (Cont.)

Singlets						Triplets				
Upper state	Dominant excitation	Coeff.	Energy (eV)	Wavelength (nm)	Oscillator strength	Upper state	Dominant excitation	Coeff.	Energy (eV)	Wavelength (nm)
	135→141	0.13725					134→140	0.1031		
	136→141	-0.17029					136→138	-0.22666		
S_{19}(A)	133→138	0.53037	4.3763	283.31	0.016		136→139	0.38232		
	133→139	0.28409					136→140	-0.38067		
	133→140	-0.14855					137→141	0.19623		
	134→138	0.19293				T_{13}(A)	133→142	0.16078	3.6553	339.19
	134→139	0.17224					134→141	-0.15432		
S_{20}(A)	132→138	-0.10758	4.4115	281.05	0.0301		134→143	0.18173		
	132→139	0.16737					135→139	0.19791		
	132→140	0.10027					135→141	-0.13468		
	133→139	0.27454					136→140	0.19818		
	133→140	0.44164					137→142	0.39589		
	135→139	-0.10295					137→143	0.20917		
	135→140	-0.14983				T_{14}(A)	133→139	0.11998	3.8054	325.81
	135→141	-0.29618					133→140	0.12606		
S_{21}(A)	133→140	0.32999	4.4647	277.7	0.0575		134→140	-0.14954		
	133→141	0.17307					135→138	-0.11204		
	134→141	-0.23081					135→139	0.18443		
	135→140	-0.10748					135→140	0.50823		
	135→141	0.48172					137→144	-0.16228		
	136→142	-0.11901				T_7(A)	132→145	-0.14173	3.843	322.62
S_{22}(A)	132→138	-0.26665	4.5113	274.83	0.0039		133→139	-0.12079		
	132→139	0.52701					133→140	0.12281		
	133→140	-0.21192					133→141	0.14379		
	136→142	-0.18505					133→142	0.1142		

Table S9. Vertical transitions, energies, and oscillator strengths of FIrpic from the S_0 state calculated using PBE0/6-311+G*/LanL2TZf. (Cont.)

Singlets						Triplets				
Upper state	Dominant excitation	Coeff.	Energy (eV)	Wavelength (nm)	Oscillator strength	Upper state	Dominant excitation	Coeff.	Energy (eV)	Wavelength (nm)
S_{23}(A)	132→139	0.14586	4.5315	273.6	0.0483		134→140	-0.27935		
	134→142	-0.1029					134→143	-0.12145		
	135→142	-0.12301					135→138	-0.10721		
	136→142	0.62501					135→139	0.17647		
	137→144	-0.12168					137→141	-0.23841		
S_{24}(A)	132→140	-0.23258	4.5955	269.8	0.1159		137→144	0.31712		
	134→140	-0.13402				T_{15}(A)	129→138	-0.15006	3.8894	318.77
	136→143	0.52061					131→138	0.47139		
	137→144	-0.29799					131→139	0.19964		
S_{25}(A)	132→138	-0.10851	4.6121	268.83	0.0638		132→138	0.19053		
	132→140	0.57044					134→138	-0.18232		
	133→139	-0.12426					137→141	-0.10616		
	133→140	-0.13415				T_{16}(A)	132→145	-0.11306	3.9221	316.11
	136→143	0.27544					134→140	-0.16759		
S_{26}(A)	131→138	-0.18948	4.705	263.52	0.0875		135→138	0.16994		
	132→138	0.36031					137→141	0.2924		
	132→139	0.20312					137→142	-0.1709		
	135→142	-0.23713					137→143	0.29537		
	136→143	0.16142					137→144	0.15745		
	137→144	0.32925					137→145	0.24682		
	137→145	0.11844				T_{17}(A)	132→144	-0.2287	3.9434	314.41
S_{27}(A)	131→138	-0.18285	4.7118	263.13	0.0626		133→138	-0.10243		
	132→138	0.38466					133→139	0.14324		
	132→139	0.20697					133→140	-0.10078		
	132→140	0.18858					134→138	0.14061		

Table S9. Vertical transitions, energies, and oscillator strengths of FIrpic from the S_0 state calculated using PBE0/6-311+G*/LanL2TZf. (Cont.)

Singlets						Triplets				
Upper state	Dominant excitation	Coeff.	Energy (eV)	Wavelength (nm)	Oscillator strength	Upper state	Dominant excitation	Coeff.	Energy (eV)	Wavelength (nm)
	134→141	-0.18456					134→139	-0.19876		
	135→142	0.18986					137→141	-0.15859		
	136→143	-0.12024					137→142	0.17617		
	137→144	-0.25423					137→144	-0.17011		
	137→145	-0.10754					137→145	0.35083		
S_{28}(A)	133→143	0.18409	4.745	261.3	0.0286	T_{18}(A)	128→138	-0.13312	3.9581	313.24
	134→143	-0.24307					129→138	-0.20046		
	135→143	0.58284					129→141	0.11475		
S_{29}(A)	132→139	0.15299	4.7507	260.98	0.0442		130→138	-0.25062		
	133→141	-0.19272					130→139	-0.10703		
	134→141	0.53453					130→141	0.11371		
	135→141	0.25889					131→138	-0.25066		
	135→142	0.12404					131→139	-0.11062		
	136→141	0.13757					132→138	-0.12042		
S_{30}(A)	132→140	-0.1214	4.7963	258.5	0.3278		135→138	0.31783		
	133→139	-0.13822					135→139	0.14031		
	133→142	0.14525					137→141	-0.14004		
	134→142	-0.2283					137→143	-0.11037		
	135→142	0.46163								
	135→143	-0.14224								
	137→144	0.25769								

Table S10. Vertical transition energies from the optimized S_0 structure of FIrpic to the T_n states, and the energy differences between T_n and T_1, calculated using PBE0/6-311+G*/LanL2TZf.

Upper state	Vertical transition energy T_n–S_0 (eV)	Energy of T_n–T_1 (cm^{-1})
T_1(A)	2.7487	-
T_2(A)	2.7888	323
T_3(A)	3.1559	3307
T_4(A)	3.1662	3388
T_5(A)	3.1963	3549
T_6(A)	3.3259	4678
T_7(A)	3.4443	5565
T_8(A)	3.4767	5888
T_9(A)	3.5158	6211
T_{10}(A)	3.5690	6614
T_{11}(A)	3.6208	7017
T_{12}(A)	3.6553	7340
T_{13}(A)	3.8054	855
T_{14}(A)	3.8430	8791
T_{15}(A)	3.8894	9195
T_{16}(A)	3.9221	9437
T_{17}(A)	3.9434	9598
T_{18}(A)	3.9581	9759

Table S11. Vertical transitions, energies, and oscillator strengths from the optimized T_1 structure of FIrpic to the T_n states calculated using PBE0/6-311+G*/LanL2TZf.

Triplets					
Upper state	Dominant excitation	Coeff.	Energy(eV)	Wavelength (nm)	Oscillator strength
T_2(A)	138A→140A	-0.11839	0.5957	2081.39	0.0155
	134B→137B	0.38129			
	136B→137B	0.90042			
T_3(A)	138A→139A	0.87165	0.8504	1457.92	0.0002
	138A→140A	-0.28958			
	138A→143A	0.10481			
	133B→137B	-0.21468			
	134B→137B	-0.10335			
	135B→137B	0.28616			
T_4(A)	138A→139A	-0.35642	0.8602	1441.41	0.0003
	133B→137B	-0.50729			
	135B→137B	0.75393			
T_5(A)	138A→139A	0.32278	0.9215	1345.53	0.0012
	138A→140A	0.87751			
	134B→137B	0.33006			
T_6(A)	138A→140A	-0.34368	1.0024	1236.88	0.0328
	138A→141A	0.12933			
	138A→143A	-0.30494			
	132B→137B	-0.10377			
	133B→137B	0.13164			
	134B→137B	0.7704			
	136B→137B	-0.35119			
T_7(A)	138A→141A	-0.62004	1.26	983.98	0.0114
	138A→142A	0.10057			
	138A→143A	0.68332			
	134B→137B	0.26924			
	136B→137B	-0.1455			
